# Comparative Phenotypic, Genomic, and Transcriptomic Analyses of Two Contrasting Strains of the Plant Beneficial Fungus *Trichoderma virens*

**DOI:** 10.1128/spectrum.03024-22

**Published:** 2023-01-31

**Authors:** Shikha Pachauri, Rinat Zaid, Pramod D. Sherkhane, Jamela Easa, Ada Viterbo, Ilan Chet, Benjamin A. Horwitz, Prasun K. Mukherjee

**Affiliations:** a Nuclear Agriculture and Biotechnology Division, Bhabha Atomic Research Centre, Trombay, Mumbai, India; b Homi Bhabha National Institute, Anushaktinagar, Mumbai, India; c Faculty of Biology, The Technion – Israel Institute of Technology, Haifa, Israel; d Robert H. Smith Faculty of Agriculture, Food and Environment, The Hebrew University of Jerusalem, Rehovot, Israel; Universidade de Sao Paulo

**Keywords:** *Trichoderma*, biocontrol, comparative genomics, mycoparasitism, induced resistance, nanopore sequencing, CEL-RNA-Seq, fungal-plant interaction, genomics, mycotrophy, transcriptomics

## Abstract

Trichoderma virens is a beneficial fungus that helps plants fight pathogens and abiotic stresses and thereby enhances crop yields. Unlike other *Trichoderma* spp., there are two well-defined strains (P and Q) of T. virens, classified by secondary metabolites profiling, primarily the biosynthesis of the nonribosomal, strong antimicrobial agents gliotoxin (Q) and gliovirin (P). We have studied the phenotypic and biocontrol properties of two well-studied representative isolates (*T. virens* Gv29-8 and *T. virens* GvW/IMI304061) that represent a Q strain and a P strain of *T. virens*, respectively. We refined the genome assembly of the P strain using nanopore technology, and we compared it with the Q strain. The differences between the genomes include gene expansion in the Q strain. *T. virens* Gv29-8 is weaker than GvW as a mycoparasite on the broad host-range plant pathogen Sclerotium rolfsii, and it is ineffective as a biocontrol agent when applied to pathogen-infested soil. *T. virens* Gv29-8 proved to be phytotoxic to *Arabidopsis* seedlings, whereas the effect of *T. virens* GvW was not major. Both strains colonized the surface and outer cortex layer of tomato roots, with about 40% higher colonization by *T. virens* Gv29-8. *T. virens* Gv29-8 induced the expression of a larger set of tomato genes than did *T. virens* GvW, although some tomato genes were uniquely induced in response to *T. virens* GvW. We studied the comparative transcriptome response of *T. virens* Gv29-8 and *T. virens* GvW to S. rolfsii. A larger set of genes was regulated in *T. virens* GvW than in *T. virens* Gv29-8 in the presence of the plant pathogen.

**IMPORTANCE** *Trichoderma virens* populations that were earlier classified into two strains (P and Q) based on secondary metabolites profiling are also phenotypically and genetically distinct, with the latter being ineffective in controlling the devastating, broad host range plant pathogen *Sclerotium rolfsii*. The two strains also provoke distinct as well as overlapping transcriptional responses to the presence of the plant and the pathogen. This study enriches our knowledge of *Trichoderma*-plant-pathogen interactions and identifies novel candidate genes for further research and deployment in agriculture.

## INTRODUCTION

*Trichoderma virens* is one of the most researched and commercially used species in the *Trichoderma* genus. This interest stems from the plant beneficial effects of *T. virens*, owing to its abilities to parasitize many pathogenic fungi, to inhibit microbes through the production of secondary metabolites, to induce plant defense, and to promote plant growth. This species is also amicable to genetic manipulation, facilitating research on basic biology. Unlike more than 300 *Trichoderma* species that have been described so far (1), *T. virens* is unique, as this species is comprised of two well-defined groups of strains. Based on their ability to synthesize major secondary metabolites, Howell and his group classified a collection of 132 isolates into two groups: “P” strains, which produce gliovirin but not gliotoxin, and “Q” strains, which produce gliotoxin and not gliovirin (2). Both of the strains produce the steroidal antibiotic viridin and its reduced form viridiol (2). One strain from each of these two groups has been studied extensively as genetic tool for biocontrol research: *T. virens* Gv29-8 (Q strain) and *T. virens* GvW/IMI 304061 (P strain) (3–14). The genome of *T. virens* Gv29-8 was sequenced and analyzed, and using a bioinformatics approach, the gliotoxin cluster was identified (15, 16). Later, using a gene knockout approach, the role of this NRPS (nonribosomal peptide synthetase) cluster in gliotoxin biosynthesis could be ascertained (13). Considerable effort and resources are devoted to the sequencing of *Trichoderma* genomes (Table S1). The systematic comparison of the P and Q strains began when we earlier reported a draft genome (NGS) assembly of a “P” strain (*T. virens* GvW) (14). The genome, sequenced using an Illumina platform, could be assembled into 107 scaffolds (compared to 135 scaffolds in the *T. virens* Gv29-8 strain “version 1” genome assembly and 93 in “version 2”). Mining the earlier version of our “P” strain genome, we identified a strain-specific NRPS cluster and established its role in gliovirin biosynthesis via gene knockout (14). Additionally, in the same genome sequence, we could identify the viridin biosynthesis gene (17). This gene cluster was dispersed through scaffolds 59, 31, 16, 45, 20, and 25 in the *T. virens* Gv29-8 genome. Here, in the current study, we deployed a nanopore-based sequencing platform to refine and improve the assembly of the *T. virens* IMI 304061 (hereafter to as *T. virens* GvW) genome. In order to gain further insights into the differences between these two strains at the genome level, we compared these two genome assemblies. Since there have been no comparative phenotypic studies on these two strains (except for secondary metabolite profiling), we studied the developmental phenotypes, mycoparasitic behavior, biocontrol potential, and interaction with the plant hosts of *T. virens* Gv29-8 *vis-à-vis T. virens* GvW. In addition, we studied the differential transcriptome responses of these two strains while in interaction with a broad host range plant pathogen, namely, *Sclerotium rolfsii*, against which the strains behave differently during mycoparasitic interactions.

## RESULTS

### Growth and morphology.

**FIG 1:**
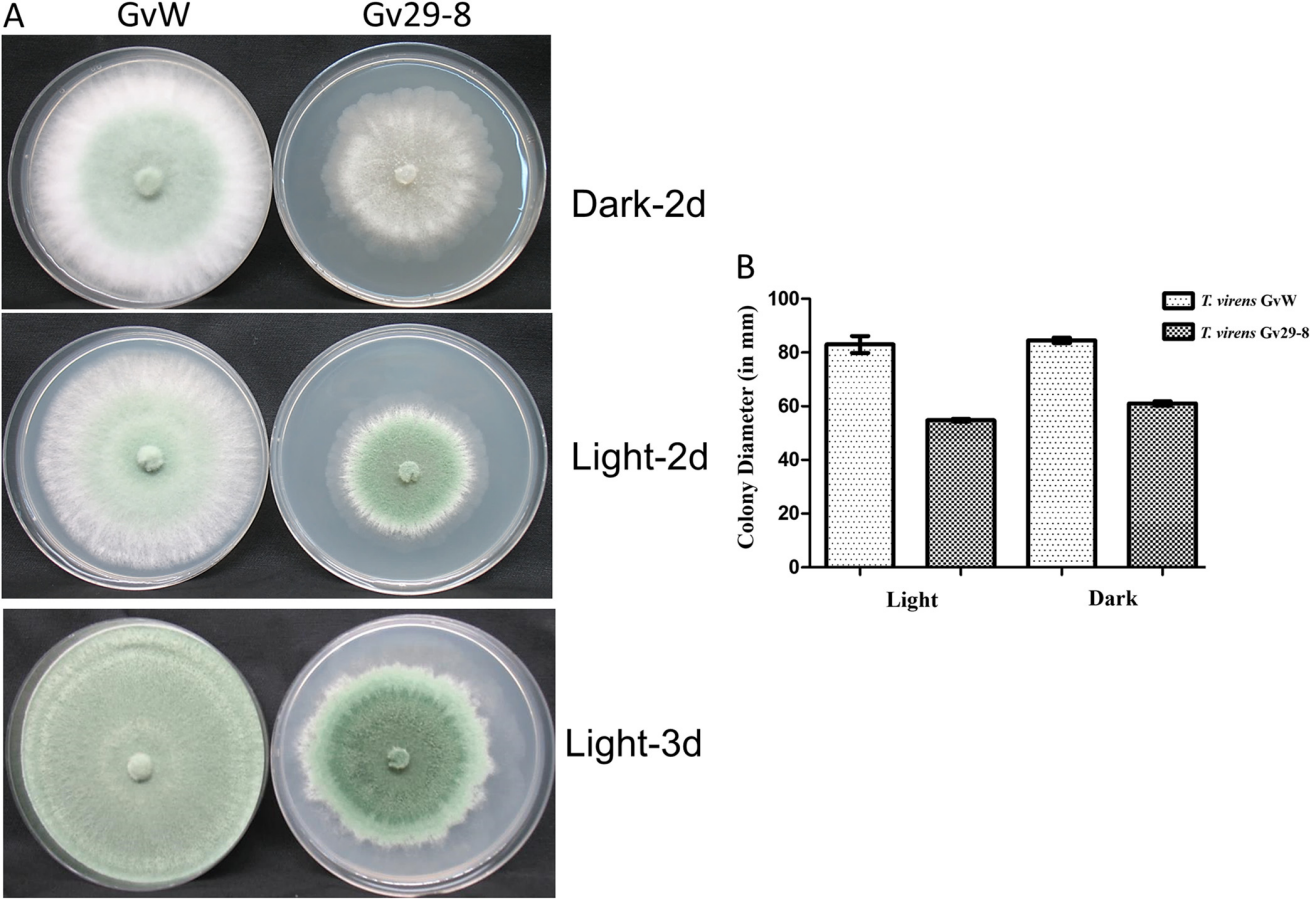
(A) Colony morphology in light and dark. Inset, the two strains were inoculated on PDA plates and grown for the indicated times in light or dark. Inset, radial growth after 2 days under light or dark. (B) The bars indicate the means of four replicate colonies.

The *T. virens* GvW strain is a fast-growing isolate, compared to the *T. virens* Gv29-8 strain (Fig. 1). After 2 days of incubation, *T. virens* GvW attained a colony diameter of 83 ± 3 mm, compared to 54.75 ± 0.5 mm in *T. virens* Gv29-8, when grown under continuous light. When incubated in the dark, after 2 days, *T. virens* GvW attained a radial growth of 84.5 ± 1 mm, compared to 61 ± 0.8 mm in *T. virens* Gv29-8. The colonies also had distinct morphologies, depending on the strain. The *T. virens* GvW strain started sporulating in 2 days, even in the dark, whereas *T. virens* Gv29-8 did not sporulate in this time. When grown on water agar, the *T. virens* GvW strain produced conidiophores in a dispersed manner, compared to the aggregates of conidiophores in the case of *T. virens* Gv29-8 (Fig. S1).

### Confrontation assay.

**FIG 2:**
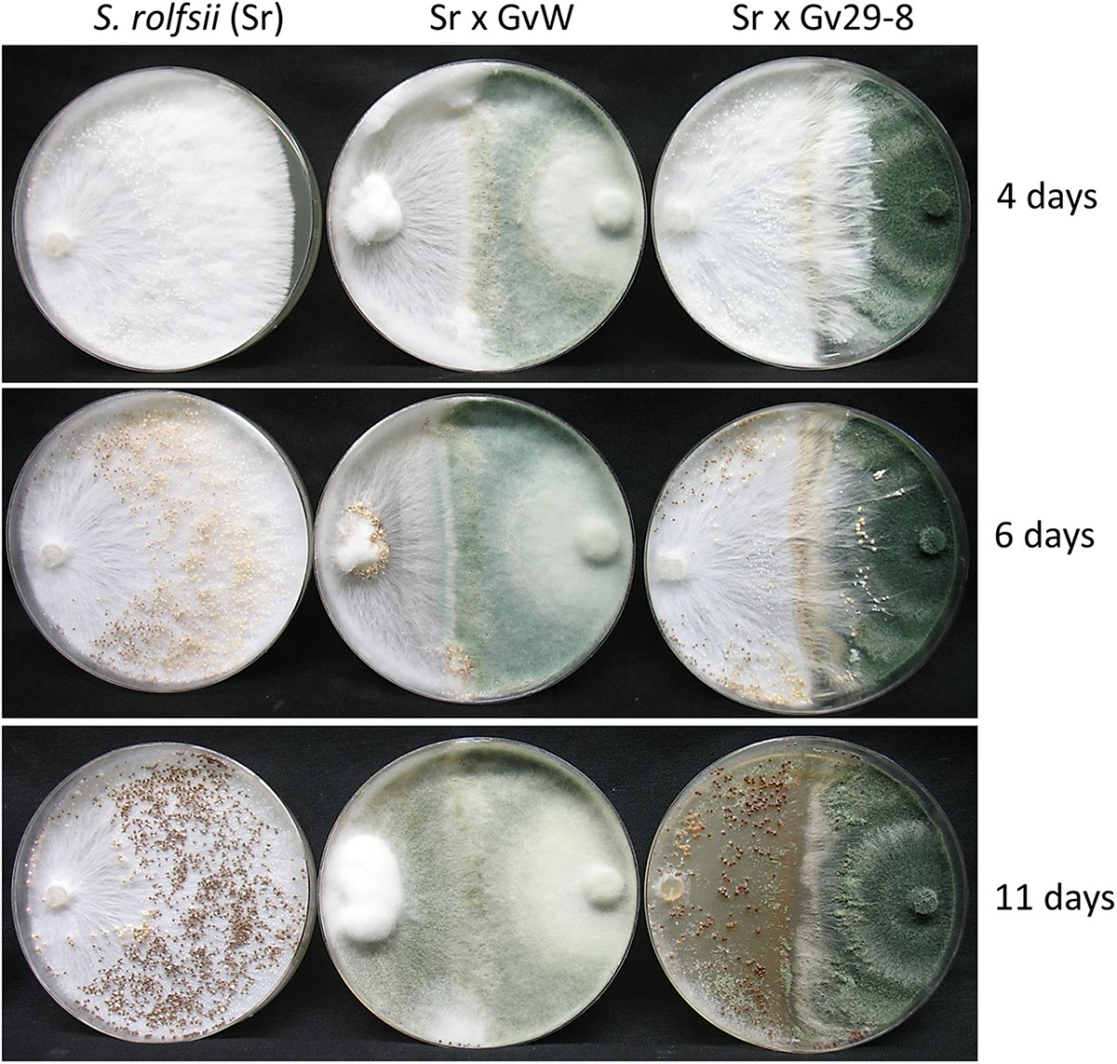
Confrontation assays. Colonies were grown for the indicated times on PDA plates. Left column, *S. rolfsii* (Sr) alone; center, confrontation with the *T. virens* GvW (Sr x *T. virens* GvW); right, *T. virens* Gv29-8 (Sr x *T. virens* Gv29-8).

In the confrontation plate assay, the two strains behaved differently when paired against *S. rolfsii*. *T. virens* GvW gradually overgrew the *S. rolfsii* colony, but initially, *S. rolfsii* started to grow over the *T. virens* Gv29-8 colony (Fig. 2). *T. virens* Gv29-8 eventually stopped the aggression of *S. rolfsii*. After 11 days of coculturing, sparse sporulation (compared to dense growth in the case of GvW) of *T. virens* Gv29-8 could be seen on the *S. rolfsii* side of the confrontation plate. When discs from the *S. rolfsii* side of the confrontation plate were seeded on potato dextrose agar (PDA) amended with benomyl, no growth of *S. rolfsii* could be seen from either the *S. rolfsii* × *T. virens* Gv29-8 plates or the *S. rolfsii* × *T. virens* GvW plate, indicating the killing of *S. rolfsii* by both of the strains (Fig. S2).

### Parasitism of sclerotia and biocontrol in soil.

**FIG 3:**
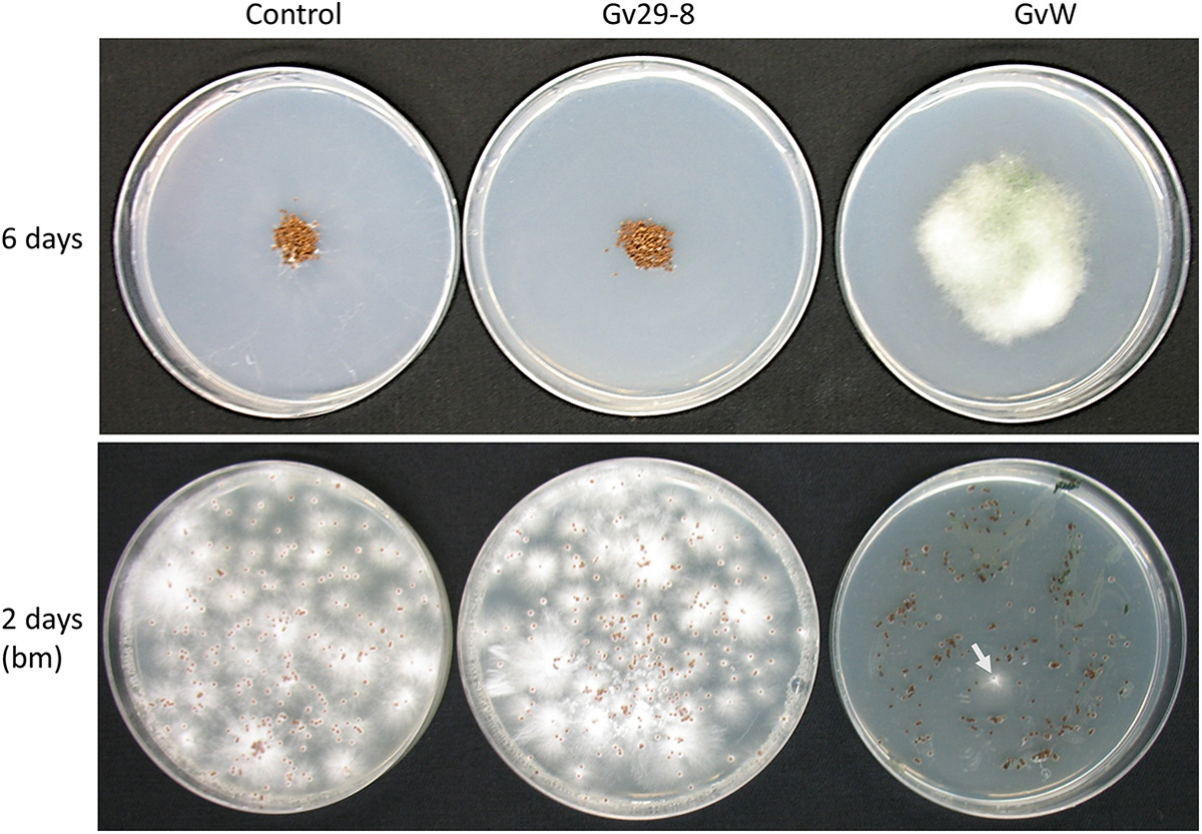
Parasitism of sclerotia. Top row, *S. rolfsii* sclerotia were plated on water agar plates: control (left), preseeded with conidia of the *T. virens* Gv29-8 strain (center), or preseeded with conidia of the *T. virens* GvW strain (right). At day 6, the sclerotia were collected, plated for revival on benomyl 10 ppm (bm), which prevents the growth of *T. virens* but permits the growth of *S. rolfsii*, and photographed 2 days later. Left and center, sclerotia appear fully viable; right, one sclerotium germinated (arrow).

On a water agar plate, all of the sclerotia in the control germinated, but they were inhibited in plates that were preseeded with the *Trichoderma* strains (Fig. 3; Fig. S3). The *T. virens* GvW strain readily colonized the sclerotia of *S. rolfsii* with growth of *Trichoderma* visible within 3 days of incubation, and, by the sixth day, profuse growth of the *T. virens* GvW strain was seen on the sclerotia, unlike *T. virens* Gv29-8 (Fig. 3). When transferred to selective medium (PDA amended with benomyl), all of the sclerotia from the control and *T. virens* Gv29-8 plates germinated, whereas only one sclerotium germinated from the *T. virens* GvW-inoculated plates (Fig. 3). In contrast to live sclerotia, dead sclerotia could be colonized by *T. virens* Gv29-8, although the extent of growth of the *T. virens* GvW strain was higher, even on dead sclerotia (Fig. S3). Consistent with its greater activity in plate confrontation assays and its parasitism of sclerotia, *T. virens* GvW protected bean seedlings grown in *S. rolfsii*-infested soil, whereas *T. virens* Gv29-8 could not (Fig. S4).

### Plant growth, root colonization, and defense response.

**FIG 4:**
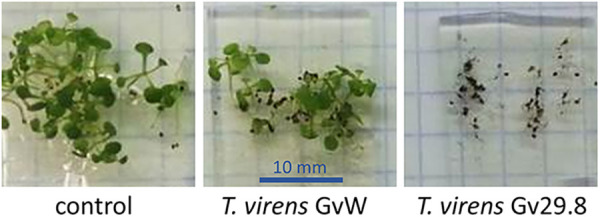
*T. virens* Gv29-8 overgrows *Arabidopsis* seedlings in a plate assay. 2.5 × 10^4^ conidia/plate of *T. virens* Gv29-8 were applied to the bases of the plants, and the plate was photographed 2 weeks postinfection.

**FIG 5:**
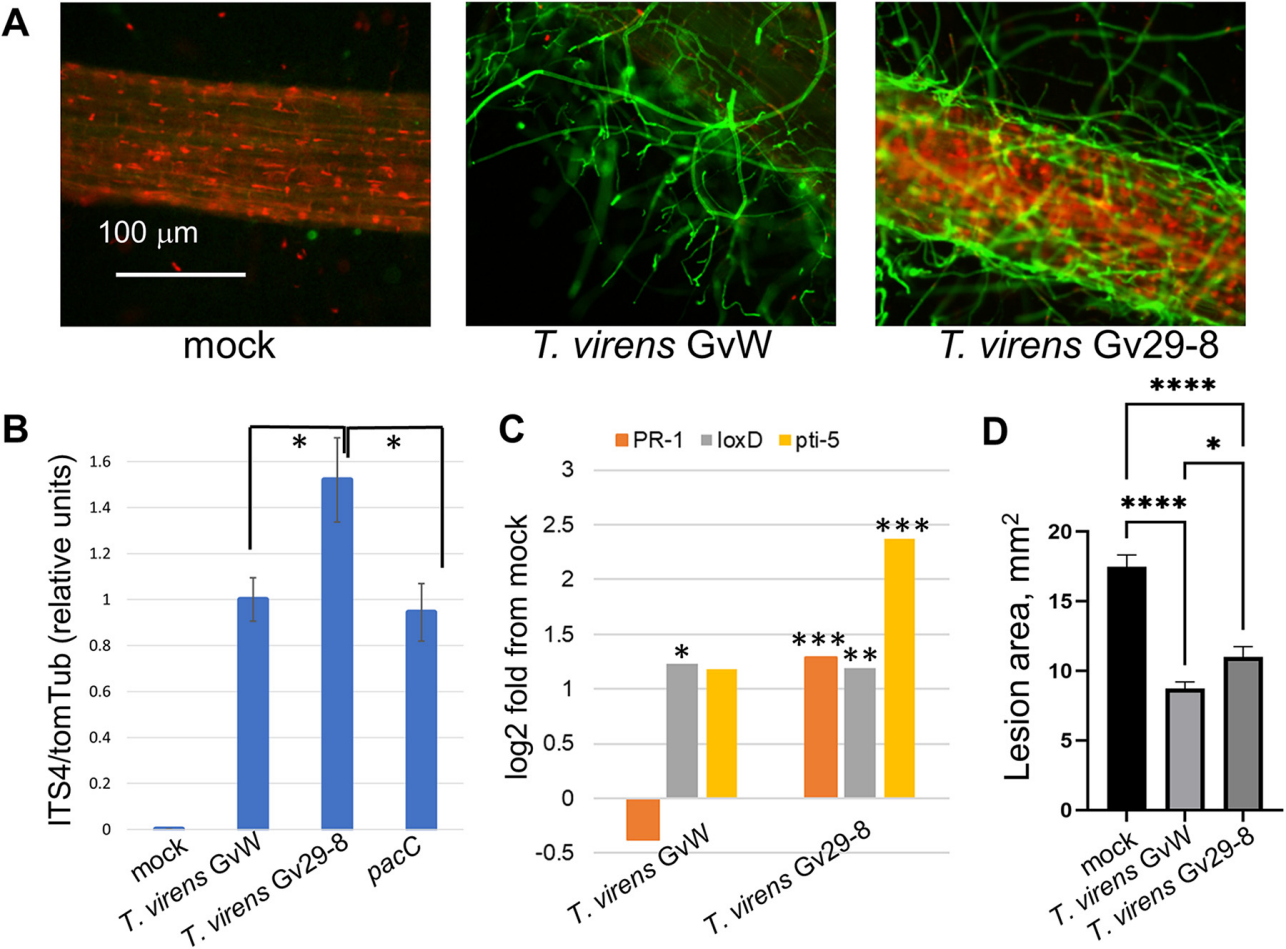
Root colonization and plant response. (A) Confocal microscopy images of tomato roots colonized by *T. virens*. Hyphae were visualized via staining with WGA Alexa-fluor (green channel). Propidium iodide fluorescence (red channel) was used to visualize the plant cell walls and nuclei. (B) Colonization assay, tomato. The *y* axis indicates the ratio of fungal to plant qPCR amplification, normalized to the value for the *T. virens* GvW (P) strain in each experiment. TomTub, tomato tubulin primers; ITS4, fungal ITS primers (see Materials and Methods). Samples from a mock inoculation with water show the background signal obtained with ITS primers. A mutant in the pH-sensitive transcriptional regulator PacC (8) in the *T. virens* GvW background is shown for comparison. The bars indicate the mean and SEM of six to nine samples from a total of three independent experiments. An asterisk indicates that a difference is statistically significant with *P* < 0.05 (two-tailed *t* test). (C) Transcript levels of the tomato genes PR1, Lox-D, and Pti-5 from the RNA-Seq data (19). The libraries were prepared from total RNA extracted from seedling shoots collected at 4 dpi (19). The bars indicate the mean and SEM of the normalized reads abundance for *n* = 4 biological repeats per treatment, An asterisk indicates a statistically significant difference from the mock with *P* < 0.05 (two-tailed *t* test). (D) Induced systemic resistance in a maize assay. Germlings from an overnight shake culture of each *Trichoderma* strain in potato dextrose medium (1.5 mL of germlings pelleted at 3,900 × *g* for 5 min) were inoculated into the medium bathing the roots of 7-day-old maize seedlings, cultured for an additional 4 days, and challenged via the drop inoculation of *Cochliobolus heterostrophus* conidia on the leaves (5, 34). The means and standard errors (*n* = 117 to 176 individual lesions from 3 independent experiments) are plotted. The means were compared via a non-Gaussian, ANOVA-like analysis, using the false discovery rate correction for multiple comparisons (35). The calculations were performed and the plot was generated using GraphPad Prism 9. The lesion sizes for the root interaction with the *T. virens* GvW and *T. virens* Gv29-8 strains differ from those of the controls, with an adjusted *P* value of <0.0001 (indicated by ****), and *T. virens* Gv29-8 differs from *T. virens* GvW with *P* < 0.05 (indicated by *).

When *T. virens* Gv29-8 conidia were directly inoculated onto the bases of *Arabidopsis* seedlings growing on 0.5 × MS agar plates, the fungus overgrew the seedlings and destroyed them, whereas *T. virens* GvW had no major effect, despite the configuration, which gave a maximum inoculum (Fig. 4). *Arabidopsis* seedlings are exceedingly small and sensitive. So, further comparisons of the P and Q strains were done with tomato seedlings. *T. virens* Gv29-8 also inhibited tomato seedling growth to some extent (18, 19). Both strains colonized tomato roots (Fig. 5A). The propidium iodide staining of nuclei indicates permeable, usually dead, cells. Here, some cell death is visible; however, most of the staining is localized to the root hair and cell walls, rather than to the nuclei of internal root tissues (Fig. 5A). In quantitative colonization assays, *T. virens* Gv29-8 (Q strain) is a significantly more aggressive colonizer of tomato roots, compared to *T. virens* GvW (P strain) (Fig. 5B). In contrast, a mutant in the pH-sensitive transcriptional regulator PacC in the GvW strain background showed no significant difference from wild type GvW. Although this is a negative result from the point of view of understanding the factors that affect colonization, the *pacC* data serve here to strengthen the conclusion that the 50% higher number in Gv29-8 is not a spurious difference, keeping in mind that colonization data are never completely quantitative (Fig. 5B). The transcriptional response of tomato seedling shoots to root interactions with the *T. virens* Gv29-8 and *T. virens* GvW strains was compared via an analysis of the transcript abundance of three known reporters of the tomato defense response that were available from the transcriptomic data obtained for our previous study (19). *Trichoderma* and gliotoxin-dependent changes in the expression of these three genes were previously studied using RNA-Seq and validated using qRT-PCR (19). PR-1 is not differentially expressed when the plant is exposed to strain GvW, whereas it is clearly induced when using Gv29-8. Both strains induced the expression of LoxD, whereas only Gv29-8 significantly induced the expression of Pti-5 (Fig. 5C). In a maize assay for induced systemic resistance (ISR), a suspension of *T. virens* germlings was inoculated into the culture medium bathing the roots of the seedlings in hydroponic culture. After coculture, the leaves were challenged with the maize pathogen Cochliobolus heterostrophus. An interaction with either *T. virens* strain significantly protected the leaves, resulting in smaller lesions. There was a small but significant (*P* < 0.05) difference between the *T. virens* Gv29-8 and *T. virens* GvW strains, with root interactions with *T. virens* GvW being slightly more effective (Fig. 5D).

To determine whether the global transcriptomic signatures of the tomato responses to the two strains are different, we compiled two gene lists from the transcriptomic data sets of (19). With the aim of finding a robust differentially regulated gene (DEG) list characterizing the two strains, the data were filtered as follows: list 1 (Table S2), transcripts significantly differential, compared to the control (mock) in interactions with both *T. virens* Gv29-8 and *T. virens* GvW, log_2_-fold change of at least 2.0 in interactions with Gv29-8; and list 2 (Table S3), significantly differential between *T. virens* Gv29-8 and *T. virens* GvW, a log_2_-fold ratio of at least 2.0 between Gv29-8 and GvW. As an example, we considered a panel of three previously studied tomato genes (20). PR-1 (Solyc01g106620) is not differentially expressed when the plant is exposed to strain GvW, although it is clearly induced when in root interaction with Gv29-8 (Fig. 5C), as confirmed via qRT-PCR (20). The other two genes in Fig. 5C are upregulated by either or both strains: LoxD (Solyc03g122340) is upregulated in both *T. virens* Gv29-8 and *T. virens* GvW, and pti-5 (Solyc02g077370) is upregulated by *T. virens* Gv29-8 but is not significantly upregulated by *T. virens* GvW. List 1 contains a total of 143 genes that were significantly upregulated or downregulated in interactions with both strains and had a log_2_-fold change of at least 2 (fourfold) in interactions with Gv29-8, perhaps representing a core of significant DEGs for interactions of tomato roots with *T. virens*. List 1 (Table S2) includes a number of annotations from the Sol tomato genome project (https://solgenomics.net/search/locus) that indicate hormone-regulated, defense-related, and transcription factor genes. With few exceptions, the pattern in this set is consistent with a qualitatively similar, but stronger, effect of *T. virens* Gv29-8. This is illustrated by the yellow (up) to violet (down) color scales in Table S3. Looking at transcripts unique to interaction with one strain only (List 2) (Table S3), 73 genes had higher transcript levels in interactions with *T. virens* Gv29-8 (violet), whereas 6 were stronger in interactions with *T. virens* GvW (yellow).

### Genome assembly and annotation.

**FIG 6:**
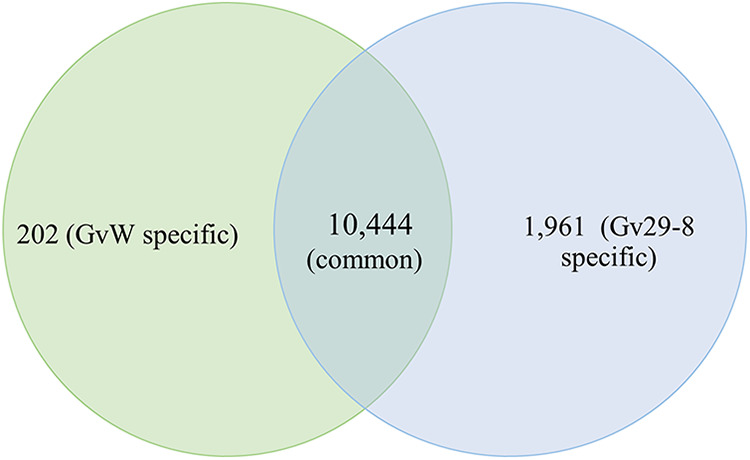
Venn diagram depicting the numbers of genes that are specific and common to the *T. virens* GvW (P) and *T. virens* Gv29-8 (Q) strains.

A total of 1.6 Gb of data were generated from the whole-genome library, with an average read length of 5.4 kb. A maximum read length of 120 kb was obtained on a Nanopore sequencer. For the *de novo* hybrid assembly, the Nanopore and Pac Bio reads were considered for the long read data, while Illumina reads were considered for the short read data. Using hybrid assembly, we could generate a high-quality genome assembly for the *T. virens* GvW strain, and it was better than many of the *Trichoderma* genome assemblies that are available in the public database, including the earlier version of the *T. virens* GvW genome assembly or version 2 of the *T. virens* Gv29-8 genome (Table S1). In the current assembly, the *de novo* whole-genome assembly size has been calculated to be 38.2 Mb, encoding 11,113 genes, of which 10,981 were predicted to be protein-coding (Table S4). The genome showed more than 98% identity with the *T. virens* Gv29-8 genome, and it was validated using a BLAST alignment against a nonredundant database (nr). A comparative analysis of *T. virens* GvW and *T. virens* Gv29-8 showed the presence of 202 proteins that are specific to the *T. virens* GvW strain and 1,961 proteins that are specific to the *T. virens* Gv29-8 strain (Fig. 6; Table S5). There are 10,444 proteins that are common between both of the strains and have unique Trividraft IDs (Fig. 6). A total of 202 proteins that are specific to *T. virens* GvW were obtained from the *de novo* assembly. These proteins show no similarity or less than 50% identity with the *T. virens* Gv29-8 genome. However, the identification of the conserved domains using NCBI-CDD and annotation using UniProtKB of these proteins revealed that 137 of these 202 proteins have predicted functional similarity to other *Trichoderma* species (Fig. S6). 1961 proteins that are specific to the *T. virens* Gv29-8 strain were obtained after subtracting the 10,444 common proteins that are found in both of the strains from the 12,405 proteins that were obtained from *T. virens* Gv29-8 genome. BLASTP searches of a random sample of 46 test sequences taken from the 1961 Gv29-8 genes that were not found in GvW identified (at cutoffs of >50% coverage and >50% identity) orthologs in other *Trichoderma* species for 67% of these protein sequences. A similar frequency, 68%, was observed for the predicted products of a sample of 32 GvW unique genes. As a control, the same analysis of a random sample of 50 test sequences from the genes shared between the two strains gave 96% with predicted orthologs in other *Trichoderma* species. Thus, both sets of strain-unique genes appear to be relatively less well-conserved than are the shared ones. More than 71% and 72% of the genes for “hypothetical proteins” and “general function” were specific to the *T. virens* Gv29-8 strain and the *T. virens* GvW strain, respectively (Tables S4 and S5; Fig. S5 and S7). Apart from genes for hypothetical proteins and general function, the highest number of genes specific to the *T. virens* Gv29-8 strain belonged to the oxidoreductase, hydrolase, and transcription factors groups, whereas the maximum number of *T. virens* GvW strain-specific genes belonged to the transferase, oxidoreductase, and hydrolase groups. The *T. virens* Gv29-8 strain showed the presence of 64 strain specific transporters, while no *T. virens* GvW strain-specific transporters were detected in the present analysis (Fig. S5 and S7). Significantly high number of genes for transcription factors, cytochrome P450, peptidase, ankyrin repeat, ribosomal protein, kinase, and HET were specific to the *T. virens* Gv29-8 strain, compared to the *T. virens* GvW strain (Fig. S5 and S7). Among the secondary metabolism-associated genes, two terpene cyclases, two polyketide-cyclase-like genes, and six NRPS genes were *T. virens* Gv29-8 strain-specific whereas two NRPS and five polyketide synthases were *T. virens* GvW strain-specific (Fig. S5 and S7).

### Transcriptome in interaction with a soilborne plant pathogen.

**FIG 7:**
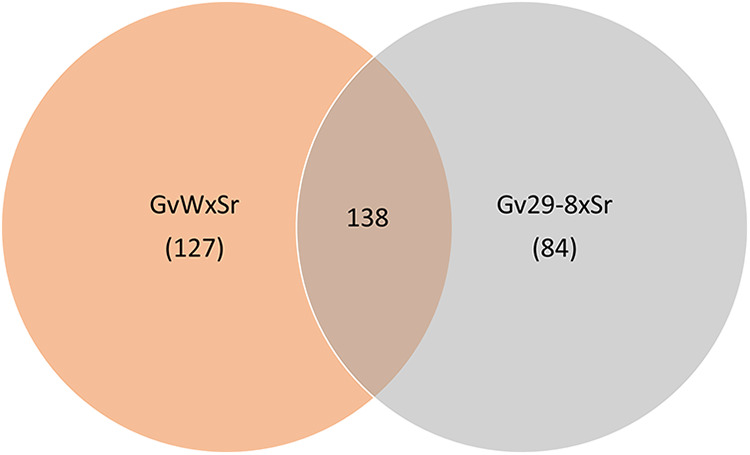
Venn diagram depicting the number of genes that are upregulated in the confrontation between *S. rolfsii* and the *T. virens* GvW (P) and *T. virens* Gv29-8 (Q) strains.

Samples of *T. virens* GvW and *T. virens* Gv29-8 mycelia from the early zone of interaction after 2 days of growth with the soilborne plant pathogen *S. rolfsii* were collected for transcriptomic analysis. The mapping of the reads to the reference genome Gv29-8 (thus considering the genes shared between the two strains) uncovered a distinct but also overlapping response to the presence of the pathogen (Table S9; Fig. 7). Choosing a cutoff of a log_2_-fold change of 2, we found that as many as 127 genes were preferentially upregulated in *T. virens* GvW interacting with *S. rolfsii*, whereas this figure was 84 in the case of *T. virens* Gv29-8; 138 regulated transcripts were common to both at the chosen cutoff level (Fig. 7; Tables S6 and S7). Of the 20 most strongly upregulated transcripts in each of the interactions of GvW and Gv29-8 with *S. rolfsii*, only 4 are common to the two lists, with the majority of the “top 20” being significantly differential in a direct comparison of the two interactions (asterisks above the bars in Fig. S10A and B; see “Ps versus Qs” in Table S9 for the adjusted probability values). No members of the two “top 20” sets showed a significant difference in expression between GvW and Gv29-8 in the control self-interactions (Tables S8 and S9). Therefore, the transcriptomic signatures of the interactions of GvW and Gv29-8 with *S. rolfsii* diverge. The numbers on the *x* axes in Fig. S10A and B are JGI protein ID numbers. The available annotations are given in Table S8. These annotations are pipeline-generated and are therefore tentative, with the exception of the small secreted cysteine-rich protein (SSCP) and CAZy genes, which have been manually annotated. See the *T. virens* G29-8 v2.0 database at the JGI website (https://mycocosm.jgi.doe.gov/mycocosm/home) for further details. The protein ID numbers refer to pages of the JGI website that contain all of the information known about each gene (nucleotide sequence, intron/exon models, predicted protein sequence, homology, predicted domains, automatic and manual annotations). The hierarchical clustering of the entire transcriptomic data set (Table S9), after the exclusion of low-counts transcripts but without applying a fold-change filter, produced divergent clusters of coregulation. Examples are given in Fig. S10C for three patterns observed in interactions with *S. rolfsii*: upregulation in both GvW and Gv29-8, upregulation in GvW only, and upregulated in Gv29-8 only. Members of these clusters that are found in the two “top 20” lists are noted with diamond symbols and are color-coded for reference between panels A, B, and C of Fig. S10. The cluster analysis indicates that the shared and distinct transcriptional patterns are robust, rather than a result of any global differences in transcript levels between the two strains at the chosen sampling time. Oxidoreductases and transporters (such as MFS transporters, ABC transporters, and oligopeptide- and amino acid-transporters) were the dominant groups of genes to be upregulated in both of the strains. The numbers of CAZymes and peptidases/proteases that were upregulated were also high in both of the strains, whereas only a few of the genes related to secondary metabolism were upregulated (Fig. S8–S13). Above a cutoff of log_2_ = 2, 80 genes were downregulated in *T. virens* GvW interacting with *S. rolfsii*, whereas only 26 genes were downregulated in *T. virens* Gv29-8 while in interaction with the pathogen (Table S6 and S7). Among the secondary metabolism-related genes that were differentially upregulated (not regulated in *T. virens* Gv29-8 below the cutoff) in *T. virens* GvW in interaction with *S. rolfsii* were an NRPS, a PKS, a polyketide cyclase, two cytochrome P450s (one from the Tex14 NRPS/PKS cluster and one from the Tex10/ferricrocin cluster), an oxidoreductase from the Tex14 cluster, and a prenyl transferase from the Tex5 cluster. However, a β-lactamase, part of the Tex5 cluster, was upregulated in both of the strains in interactions with *S. rolfsii*. A Tex25 NRPS and a cytochrome P450 that were associated with this cluster were upregulated in both of the strains. Two GH18 family chitinases were upregulated in both *T. virens* GvW × *S. rolfsii* and in *T. virens* Gv29-8 × *S. rolfsii.* Interestingly, typical plant cell wall-degrading enzymes (pectate lyase and polygalacturonase) were among the genes that were induced in both of the strains in interactions with *S. rolfsii*. All five of the SSCPs that were upregulated in *T. virens* GvW × *S. rolfsii* were also upregulated in *T. virens* Gv29-8 × *S. rolfsii* (one below cutoff applied). One catalase (88881), six SSCPs, one GH64 family β-1,3 glucanase, two GH18 family chitinase domain-containing proteins, and a gene for a Tc toxin were selectively upregulated in *T. virens* Gv29-8 × *S. rolfsii* only, with no change in *T. virens* GvW × *S. rolfsii*. The Tex24 NRPS and an oxidoreductase of the Tex21 cluster were downregulated in *T. virens* GvW × *S. rolfsii*, while there was no change in expression in *T. virens* Gv29-8 × *S. rolfsii*. Interestingly, two chitinases belonging to the GH18 family and a chitosanase (GH75 family) were also downregulated in *T. virens* GvW × *S. rolfsii*, while one chitinase was downregulated in both *T. virens* GvW × *S. rolfsii* and *T. virens* Gv29-8 × *S. rolfsii*. Three SSCPs and four transcriptional regulators were selectively downregulated in *T. virens* GvW × *S. rolfsii* (and not in *T. virens* Gv29-8 × *S. rolfsii*). One PKS, two “A” domain proteins, and one transcriptional regulator were selectively downregulated in *T. virens* Gv29-8 × *S. rolfsii*. The transcriptomic data were obtained for the initial region of contact in the interaction between *T. virens* and *S. rolfsii* colonies. Time-series experiments via qRT-PCR for a small panel of upregulated genes (Fig. S11) suggest that some induction may occur prior to contact. However, this is a trend, not a significant difference. Just following contact, corresponding to the samples for the full transcriptome, expression levels increase and peak a day later. A notable exception is ID 69251, which encodes a small secreted cysteine rich protein (SSCP), which decreased at first in GvWxSr and then increased, with this behavior being observed both in the transcriptome (Fig. S10A) and via qRT-PCR (Fig. S11).

## DISCUSSION

*Trichoderma* spp. are biotechnologically significant fungi that are used widely in agriculture and industry. Previous genomic studies, especially whole-genome sequencing, have helped understand the genetic basis of many biological traits, may it be for the production of enzymes of industrial interest or for the antagonistic properties (14, 15, 17, 21, 22). Even though the genomes of several *Trichoderma* spp. and strains have been sequenced, an integrated, comparative, strain-specific study of the phenotypes *vis-à-vis* the gene expression as well as the genome sequence has been lacking. *T. virens* Gv29-8 and *T. virens* GvW (IMI 304061), representing a Q strain and a P strain, respectively, have been extensively studied for their gene functions and biocontrol potential. *T. virens* Gv29-8 is reported to be effective against R. solani, S. sclerotiorum and *Globisporangium* (*Pythium*) *ultimum*, whereas *T. virens* GvW has been reported to be active against R. solani, P. aphanidermatum and *S. rolfsii*. Here, we compare these two strains for their growth and colony morphology, mycoparasitic behavior, biocontrol potential, root colonization, and induced defense properties, along with the transcriptomic response of *T. virens* Gv29-8, a weak mycoparasite, *vis-à-vis T. virens* GvW, a strong mycoparasite on *S. rolfsii*. Finally, we compare the whole-genome sequences of these two strains in our quest for finding genetic differences that might, hypothetically, correlate with the differential behavior of these two strains. *T. virens* Gv29-8 produces copious amounts of gliotoxin and, hence, is a slow growing isolate (gliotoxin-negative mutants are fast growing) (13). *T. virens* GvW produces gliovirin, but it does so in a low quantity, and, hence, the deletion of the gliovirin coding gene does not alter the growth rate (14). Even though both of the strains are active mycoparasites on R. solani and Globisporangium/Pythium spp., our data indicate that *T. virens* Gv29-8 is a weak parasite on *S. rolfsii*, whereas, as reported earlier, *T. virens* GvW is a strong mycoparasite (and, hence, a good biocontrol agent) on this pathogen, which infects more than 500 crop plants (23). The plant assay indicated that *T. virens* Gv29-8 is more phytotoxic to *Arabidopsis* seedlings, compared to *T. virens* GvW. This could be due to the direct effect of gliotoxin on plants (19). The production of gliotoxin also correlated with the extent of the colonization of the tomato roots by these two strains. Interestingly, in a hydroponic system, both of the strains, applied as germlings to roots, are equally effective in protecting maize foliage against a necrotrophic pathogen, in contrast to tomato, in which the gliotoxin-producing *T. virens* Gv29-8 was more effective than its gliotoxin-deficient mutant and *T. virens* GvW (20). In the tomato assay, a greater number of plant genes were induced, often to a greater extent, upon the interaction of the roots with *T. virens* Gv29-8 than with *T. virens* GvW. Nevertheless, a subset of tomato transcripts, including an expansin-like protein, was unique to interactions with T*. virens* GvW (Table S3). Thus, it will be important to study the shoot/leaf transcriptomic signatures of root interactions with different *Trichoderma* species and strains in maize and in other crop plants. In interactions with *S. rolfsii*, more genes were differentially regulated in *T. virens* GvW (265 up, 80 down), compared to *T. virens* Gv29-8 (222 up, 26 down). Among the genes that responded to the presence of potential “prey” were genes associated with secondary metabolism clusters, small cysteine-rich proteins, and GH family proteins. The role of SSCPs in fungal-fungal interactions would be an interesting subject for future studies.

We have refined the genome assembly of *T. virens* GvW using a combination of short reads and long reads (Nanopore), which helped to reduce the number of scaffolds from 93 in the reference genome (*T. virens* Gv29-8) to 47 in the current version (*T. virens* GvW). A comparison of the whole-genomes indicates a genome expansion in *T. virens* Gv29-8 (39 Mb versus 38.2 Mb in *T. virens* GvW), with 202 proteins that are unique to the *T. virens* GvW strain, whereas a large set of 1,961 genes are unique to *T. virens* Gv29-8, corresponding to genome expansion. Based on multilocus phylogeny, Cai and Druzhinina (1) proposed that *T. virens* GvW could be a new species that is closer to T. neocrassum, whereas *T. virens* Gv29-8 is a confirmed *T. virens*. However, the authors could not assign a species name to IMI 304061. More analyses involving several P and Q strains would be required to reclassify *T. virens* P strains as belonging to a new species. However, even though they are morphologically similar, the large genomic differences between these two strains point to the fact that the P and Q strains of *T. virens* might be separate species. A new taxonomy needs to be proposed and accepted in order to reclassify the P strains of *T. virens* as novel species.

## MATERIALS AND METHODS

### Strains and growth conditions.

The *T. virens* GvW was isolated from agricultural soil from Pantnagar, Uttarakhand, India, using *S. rolfsii* as a bait (24). The *T. virens* Gv29-8 was originally isolated from agricultural soil from a field near College Station, Texas (25). The *S. rolfsii* isolate was from our previous study (9). The cultures were routinely grown on potato dextrose (Difco) medium with 1.5% agar (PDA) at 25 to 27°C. For long-term storage, the *Trichoderma* conidia and *S. rolfsii* sclerotia were preserved as glycerol stocks in 30% glycerol at −80°C.

### Growth and conidiation.

Mycelial discs from freshly grown cultures were inoculated at the centers of 85 mm PDA plates and were incubated at ambient temperatures (25 to 27°C) either under a fluorescent lamp or in complete darkness. The cultures were monitored for the presence or absence of conidia visually and under a microscope. For studying the conidiophore branching patterns, cultures were grown on a 1% agar plate at ambient temperature (25 to 27°C) under laboratory light and were photographed directly under a microscope after 3 days of incubation. All of the experiments were done in four replicates and were repeated twice for reproducibility.

### Confrontation assay.

To assess the ability of *T. virens* to overgrow and kill *S. rolfsii* in the confrontation assay, both of the fungi were simultaneously inoculated at opposite ends of a PDA plate and incubated at ambient temperature (25 to 27°C) under laboratory light. Periodical observations were recorded on the overgrowth of one fungus on the other. At the end of the incubation period, the growth from the zone of confrontation was transferred to PDA amended with 10 ppm benomyl (Sigma). Benomyl suppresses the growth of *T. virens*, allowing for *S. rolfsii* to grow, and this assay ascertains the viability of *S. rolfsii* in confrontation plates.

### Parasitism of sclerotia.

*S. rolfsii* produces round, mustard seed-like structures, called sclerotia, that survive in soil in the absence of active host plants and germinate and infect newly sown crop plants (26). The ability of *T. virens* to colonize and kill the sclerotia was assessed as described earlier (23). Briefly, *S. rolfsii* was cultured on PDA for 1 month to obtain fully mature sclerotia. Sclerotia were harvested with a paint brush, and about 100 sclerotia were seeded at the center of a water agar plate that was previously seeded with 100 μL of a spore suspension that contained 10^6^ conidia. The plates were incubated in a humid chamber, and observations on the growth of the *Trichoderma* on the sclerotia were recorded regularly. After 6 days, the sclerotia were put on a PDA-benomyl (10 ppm) plate for an assessment of viability. For comparison, the same experiment was set up with dead (dry autoclaved) sclerotia.

### Root colonization and effect on plant growth.

**(i) Tomato.** The colonization of the tomato roots was assayed via qPCR. Seeds of Solanum lycopersicum cultivar BQ328 (Eden Seeds, Israel) were surface sterilized with diluted bleach (final concentration 1% sodium hypochlorite in sterile deionized water) for 10 min and then washed three times in sterile water. Seeds were placed in plant culture boxes (magenta, available from Sigma-Aldrich-Merck as V8505), five seeds/box, containing sterile, half-strength (0.5×) MS medium that was adjusted to pH 5.7, with 0.8% agar (plant culture grade agar, Caisson or Duchefa) (27). After 10 days of growth at 22 to 25°C and 16/8 h light/dark cycles, they were inoculated with *Trichoderma* conidia, 5 × 10^4^ per plate in 1 mL suspension in water, applied to the bases of the plants. Three days later, the plants were gently lifted from the agar. Fungal growth and sporulation were visible on the agar surface, but this was with no visible adhering mycelium on the roots, which were frozen and ground in liquid N_2_ for genomic DNA extraction. To extract DNA,100 to 200 μg powder was vortexed in 400 μL extraction buffer (200 mM Tris HCl [pH 7.5], 25 mM EDTA, 0.5% SDS, 250 mM NaCl), incubated for 30 min at room temperature, and centrifuged for 1 min at 18,000 × *g*. An equal volume of cold isopropanol was added to 300 μL of the supernatant, mixed, and, after 2 min of incubation at room temperature, centrifuged for 5 min at 18,000 × *g*, washed with 70% ethanol, dried, and dissolved in 100 μL TE buffer. 1.5 μL per well were assayed via qPCR in 15 μL of reaction volume with PerfeCTa SYBR Mix (Quanta) in an Applied Biosystems 7300 cycler. The fungal ITS primers for *Trichoderma* were: TGP4-F 5′-CTCCCAAACCCAATGTGAAC-3′/TGP4-R 5′-GCGAGTGTGCAAACTACTG-3′ (28). Tomato β-tubulin was used as an endogenous reference gene for normalizing fungal gDNA to tomato gDNA PF: 5′-CAGTGAAACTGGAGCTGGAA-3′; PR: 5′-TATAGTGGCCACGAGCAAAG-3′ (29). The fungal ITS qPCR signal was linear, with increasing sample amounts to 500 mg fresh weight of *Trichoderma* mycelium.

**(ii) Arabidopsis.** Seeds were surface sterilized via dry sterilization. An open Eppendorf tube containing approximately 20 μL of seeds was placed in a closed chamber, inside a chemical hood, near sterilization solution (0.3% sodium hypochlorite solution supplemented with 2% hydrochloric acid) for 4 h. Sterilized seeds were aerated in a sterile hood for at least 15 min, and they were then arranged in a row on petri dishes containing 0.5× MS agar, as above for the tomato. The plates were stratified for 2 to 3 days at 4°C in the dark to promote germination, and they were then kept at 22 to 25°C with 16/8 h light/dark cycles. On the tenth day, they were inoculated with *Trichoderma* conidia, 2.5 × 10^4^ per plate, applied to the bases of the plants, and were photographed 2 weeks postinfection.

### Biocontrol and induced systemic resistance (ISR).

The ability of the *T. virens* strains to protect bean seedlings from *S. rolfsii* was assessed as described previously (9). The pathogen was inoculated at 150 mg sclerotia per kg sand, and *Trichoderma* were simultaneously inoculated as a spore suspension at a concentration of 10^7^ spores/mL/kg sand. Bean seeds were planted, and observations on the seedling mortality were recorded after 10 days. A maize seedling hydroponics assay was used to assess the protection (referred to here as ISR for simplicity, although the mode of protection may include elements of systemic acquired resistance) from leaf infection by Cochliobolus heterostrophus as described previously (5).

### Sequencing, assembly, and annotation of *T. virens* GvW.

High-quality genomic DNA of the *T. virens* GvW strain was extracted as described earlier, using the phenol chloroform method (30). DNA was treated with RNaseA, and enzymes were removed via phenol extraction and were suspended in TE buffer (pH 8). Further processing, sequencing, assembly, and annotation were done at M/S Genotypic, Bengaluru, India. Briefly, genomic DNA (1.6 μg) was purified using a Zymoclean Large Fragment DNA Recovery Kit. The high molecular weight genomic DNA (1.2 μg) was end-repaired using a NEBNext Ultra II End Repair Kit (New England Biolabs, MA, USA) and was purified using 1× AmPure beads (Beckmann Coulter, USA). This was followed by adapter ligation using NEB Blunt TA Ligase (New England Biolabs, MA, USA) at 20°C for 20 min. Purification was done using 0.4× AmPure beads (Beckmann Coulter, USA), and the sequencing library was eluted in 15 μL of elution buffer. Sequencing was performed on a GridION X5 (Oxford Nanopore Technologies, Oxford, UK), using a spotON flow cell R9.4 (FLO-MIN106) in a 48 h sequencing protocol on MinKNOW 2.1 v18.05.5. A total of 295,578 reads were generated from the whole-genome sequencing library. Nanopore raw reads (“*fast5*” format) were base-called (“*fastq*” format) using Albacore v2.3.1. A total of 1.6 Gb of data, with an average read length of 5.4 kb, was generated from the whole-genome library. A maximum read length of 120 kb was achieved on a Nanopore sequencer. The Illumina and PacBio raw data for the *T. virens* GvW were obtained from our previous assembly (accession number: LQCH00000000) (14). The Nanopore and Pac-Bio reads, along with the Illumina data, were used for *de novo* hybrid assembly using MaSuRCA, and a genome assembly of 38.2 Mb was obtained. The assembly was validated using BLASTX alignment against the “nr” database. Annotation was performed using the Augustus tool.

### Comparative analysis of *T. virens* GvW and *T. virens* Gv29-8 genomes.

The comparative analysis of *T. virens* GvW and *T. virens* Gv29-8 genomes was performed via the identification and characterization of the predicted proteins that are exclusively present in the respective strains. The *T. virens* GvW strain-specific proteins were identified from the 10,981 predicted proteins that were obtained from the *de novo* assembly of the *T. virens* GvW genome. The *T. virens* GvW strain-specific proteins are those which do not have the Trividraft ID and thus show no similarity with the *T. virens* Gv29-8 genome. Similarly, predicted proteins obtained from the *de novo* assembly of the *T. virens* GvW genome, or the Gv29-8 sequences entered into NCBI more recently (30), with a percent identity of less than 50% (51 proteins) with the *T. virens* Gv29-8 genome, were also identified as *T. virens* GvW strain specific proteins. These proteins were further analyzed for the presence of conserved domains, and they were annotated, based on the sequence similarity search. A BLAST-P search was performed between the *T. virens* GvW strain-specific proteins with no Trividraft ID and a nonredundant protein sequence database (nr), and this was in addition to a TBLASTN to the Gv29-8 v2.0 database at the JGI. The conserved domains in the proteins were identified using the NCBI Conserved Domains Database (NCBI-CDD). The proteins were annotated against the UniProt KnowledgBase (UniProtKB) database from the Universal Protein Resource (Uniprot). Finally, the *T. virens* GvW strain-specific proteins were further annotated, based on the classification of the proteins. For the identification of the proteins that were exclusively present in the *T. virens* Gv29-8 genome, the annotation file for 12,405 proteins obtained from the *T. virens* Gv29-8 genome was downloaded from the JGI Mycocosm site (31). The *T. virens* Gv29-8 strain-specific proteins were obtained after subtracting the common proteins that were found in both the *T. virens* GvW and the *T. virens* Gv29-8 genomes from the total 12,405 proteins that were found in the *T. virens* Gv29-8 genome. The *T. virens* Gv29-8 strain-specific proteins were also further characterized, based on the classification of the proteins.

### Transcriptome analysis.

*Trichoderma* strains were paired with *S. rolfsii* on PDA plates that were lined with a dialysis membrane and incubated at ambient temperature (25 to 27°C). *Trichoderma* strains placed against themselves served as controls. RNA samples from three independent experiments were analyzed for interactions. Additionally, RNA samples from two experiments were analyzed for the Gv29-8 controls, and RNA samples from four experiments were analyzed for the GvW controls. After two days, the interacting fungi met around the center of the plate. *Trichoderma* growths (about 5 mm) in contact with *S. rolfsii* colonies were harvested with a spatula and frozen immediately, before RNA extraction with TriReagent (Sigma), following the manufacturer’s protocol, except that the starting material was fungal material that had been ground in liquid N_2_. Total RNA was then column-purified from the phenolic phase of the extraction, using a Direct-zol RNA Miniprep Kit (Zymo), including an on-column DNase treatment. cDNA libraries were prepared using the CEL-Seq2 protocol (32) with minor changes. Instead of single-cells, 2 ng of purified total RNA were taken as the input for the library preparation. The CEL-Seq library was run on an Illumina NextiSeq550 instrument. The number of reads ranged from 6,571,796 to 39,502,414 per sample. The reads were mapped to the *T. virens* Gv29-8 genome (15), using Tophat2, version 2.1.0, with up to 3 mismatches allowed per read. The minimum and maximum intron sizes were set to 20 and 20,000, respectively, and an annotation file was provided to the mapper. The percentage of uniquely mapped reads ranged from 66.04% to 92.49% per sample. Only uniquely mapped reads were counted to genes, using the “HTSeq-count” package, version 0.6.1, with the “union” mode. Normalization and differential expression analyses were conducted using the DESeq2 R package, version 1.28.0. The sample preparation, sequencing, quality control, and differential expression analyses were conducted by the Technion Genomics Center at the Life Science and Engineering Interdisciplinary Research Center, Technion, Haifa, Israel. The cluster analysis (Fig. S10C) was done using Genesis at https://genome.tugraz.at/genesisclient/genesisclient_description.shtml (33).

**(i) Quantitative RT-PCR (qPCR).** For the time-series experiments, colonies were harvested at daily intervals, denoted 1 to 4. At time 1, the colonies were approximately 2.5 cm in diameter, and they were expanding but well-separated. Harvest time 2 was adjusted for the growth rate so that samples were taken at early interaction after the meeting of the pairs, corresponding to the harvest time for RNASeq. The samples for times 3 and 4 were harvested 1 and 2 days after time 2, respectively. Total RNA was extracted as for RNASeq. cDNA was prepared from 1 μg RNA samples, using a Quanta Bio qScript Reverse Transcriptase Kit in a 20 μL reaction, diluted 6-fold in pure water, and stored at –20°C for the preparation of successive 96-well qPCR plates (Applied Biosystems). 1.5 μL per well in a 15 μL volume reaction with PerfeCTa SYBR Mix (Quanta Bio) were amplified in an ABI 7300 qPCR cycler, and the transcript abundance was calculated from the Ct values (20).

### Data availability.

The Whole Genome Shotgun project has been deposited into DDBJ/ENA/GenBank under the accession number LQCH00000000. The version described in this paper is version LQCH02000000. The transcriptome data are appended in Table S9, and the RNA-Seq reads were deposited into SRA-NCBI, PRJNA914121.
