# Supplementary material for: Comparative Phenotypic, Genomic, and Transcriptomic Analyses of Two Contrasting Strains of the Plant Beneficial Fungus Trichoderma virens
Source: Microbiol Spectr. 2023 Jan 31;11(2):e03024-22. doi: 10.1128/spectrum.03024-22 (PMC10100780; doi:10.1128/spectrum.03024-22)
Supplement: Supplemental file 1 — Table S1, legends of Tables S2 to S9, and Fig. S1 to S13. Download spectrum.03024-22-s0001.pdf, PDF file, 1.2 MB [file spectrum.03024-22-s0001.pdf]

Table S1. Genome assembly statistics of *Trichoderma virens* IMI 304061, *vis-a-vis* some representative *Trichoderma* spp. genomes from the database

S.No	Species	Strain	GenBank accession number	Number of scaffolds /contigs	Sequence coverage	Sequence technology	Genome size (Mbp)	Protein coding genes	Reference
1	*Trichoderma arundinaceum*	IBT 40837	PXOA000 00000.1	1370	50x	Illumina MiSeq	36.9	10,539	(1)
2	*Trichoderma atroviride*	IMI 206040 v2.0	ABDG000 00000.2	29	8.26x	Sanger	36.1	11,863	(2)
3	*Trichoderma asperellum*	CBS 433.97 v1.0	MBGH000 00000.1	419	120x	Illumina	37.4	12,586	(3)
4	*Trichoderma citrinoviride*	TUCIM 6016 v4.0	MBDI000 00000.1	533	63.1x	Illumina; PacBio	33.2	9,737	(3)
5	*Trichoderma gamsii*	T6085	JPDN0000 0000.2	381	100x	Illumina MiSeq	37.9	10,944	(4)
6	*Trichoderma guizhouense*	NJAU 4742	LVVK000 00000.1	63	24x	454	38.3	11,255	(3)

7	*Trichoderma harzianum*	CBS 226.95	MBGI00000000.1	532	120x	Illumina	40.98	14,095	(3)
8	*Trichoderma harzianum*	TR274 v1.0	NQLC00000000.1	2282	100x	Illumina MiSeq	40.87	13,932	(5)
9	*Trichoderma hamatum*	GD12 v1.0	ANCB00000000.2	745	40.0x	Illumina HiSeq	38.4	10,520	(6)
10	*Trichoderma longibrachiatum*	ATCC 18648 v3.0	MBDJ00000000.1	130	104x	Illumina; PacBio	32.2	10,938	(3)
11	*Trichoderma parareesei*	CBS 125925 v1.0	LFMI00000000.1	885	250.5x	Illumina	32	9,062	(7)
12	*Trichoderma reesei*	QM6a v2.0	AAIL00000000.2	89	9x	Sanger	34.1	9,129	(8)
13	*Trichoderma reesei*	RUT C-30 v1.0	JABP00000000.1	182	47.6x	Illumina	32.6	9,852	(9)
14	*Trichoderma virens*	Gv29-8 v2.0	ABDF00000000.2	93	8.05x	Sanger	39	12,427	(2)
15	*Trichoderma virens*	IMI304061/ GvW	LQCH02000000	47	57x	Illumina, Nanopore and Pac-bio (De-novo hybrid assembly)	38.2	10,981	This study

16	*Trichoderma simmonsii*	GH-Sj1	CP075864.1 - CP075870.1	7	374x	Illumina NovaSeq; Oxford Nanopore GridION	40	13,120	(10)
17	*Trichoderma virens*	Gv29-8	CP071107 - CP071114	7 (sequence near-complete)	25.8x	Oxford Nanopore MiniION	41	12,263	(11)
18	*Trichoderma asperellum*	FT101	CP084943 - CP084950	7 (sequence near-complete)	59.1x	PacBio RSII	37.57	12,041	(11)
19	*Trichoderma virens*	FT-333	CP071115 - CP071122	7 (sequence near-complete)	83.7x	Oxford Nanopore MiniION	41.44	11,895	(11)
20	*Trichoderma atroviride*	P1	CP084935 - CP084942	7 (sequence near-complete)	153x	Oxford Nanopore MiniION	37.33	13,327	(11)
21	*Trichoderma semiorbis*	FJ059	JAIMJC000000000.1	7	105x	Oxford Nanopore PromethION; Illumina HiSeq	42.02	10,251	(12)

22	*Trichoderma afroharzianum*	T11_W	WUWT00 000000	6	233x	Illumina HiSeq; PacBio Sequel	41.2	11,811	(13)
23	*Trichoderma cyanodichotomus*	TW21990-1	WXUD00 000000	10	186x	PacBio RSII; Illumina HiSeq	36.1	10,168	(13)
24	*Trichoderma koningiopsis*	RA3a	JAJPEM0 00000000. 1	14 contigs	178x	Illumina; Oxford Nanopore MinION	36.53	8,951	(14)
25	*Trichoderma koningiopsis*	RA5	JAJPEL00 0000000	11 contigs	201x	Illumina; Oxford Nanopore MinION	36.48	8,964	(14)
26	*Trichoderma koningiopsis*	RA6	JAJPEK00 0000000	13 contigs	140x	Illumina; Oxford Nanopore MinION	36.47	9,124	(14)

27	*Trichoderma gracile*	HK011-1	JAINEY000000000.1	10 contigs	80x	Oxford Nanopore PromethION; Illumina HiSeq	34	10739	(12)
28	*Trichoderma asperellum*	IIRRCK1	JAIAZZ000000000.1	740	109x	Illumina HiSeq 2500	37.2	11,592	(15)
29	*Trichoderma asperellum*	IIRRCK4	JAICDU000000000.1	507	116x	Illumina Hi-seq 2500	39.9	14,174	(15)
30	*Trichoderma asperellum*	TVIIRRCK1	JAHYXG000000000.1	469	150x	Illumina HiSeq 2500	36	11,589	(15)
31	*Trichoderma asperelloides*	T203	JAJKFY000000000.1	354	100x	Illumina NovaSeq	36.2	11,165	(16)
32	*Trichoderma hamatum*	FBL 587	SEIV00000000	1,803	44x	Illumina MiSeq	38.9	10,944	(17)

33	*Trichoderma reesei*	QM6a	CP016232 – CP016238	7	81.4	Illumina; PacBio	34.9	10,877	(18)
34	*Trichoderma lixii*	MUT 3171	SESN00000000.1	2142	40x	Illumina MiSeq	40.89	11,923	(19)

References

1. Proctor RH, McCormick SP, Kim HS, Cardoza RE, Stanley AM, Lindo L, Kelly A, Brown DW, Lee T, Vaughan MM, Alexander NJ, Busman M, Gutiérrez S. 2018. Evolution of structural diversity of trichothecenes, a family of toxins produced by plant pathogenic and entomopathogenic fungi. PLoS Pathog 14:e1006946.
2. Kubicek CP, Herrera-Estrella A, Seidl-Seiboth V, Martinez DA, Druzhinina IS, Thon M, Zeilinger S, Casas-Flores S, Horwitz BA, Mukherjee PK, Mukherjee M, Kredics L, Alcaraz LD, Aerts A, Antal Z, Atanasova L, Cervantes-Badillo MG, Challacombe J, Chertkov O, McCluskey K, Coulpier F, Deshpande N, von Döhren H, Ebbole DJ, Esquivel-Naranjo EU, Fekete E, Flipphi M, Glaser F, Gómez-Rodríguez EY, Gruber S, Han C, Henrissat B, Hermosa R, Hernández-Oñate M, Karaffa L, Kosti I, le Crom S, Lindquist E, Lucas S, Lübeck M, Lübeck PS, Margeot A, Metz B, Misra M, Nevalainen H, Omann M, Packer N, Perrone G, Uresti-Rivera EE, Salamov A, Schmoll M, Seiboth B, Shapiro H, Sukno S, Tamayo-Ramos JA, Tisch D, Wiest A, Wilkinson HH, Zhang M, Coutinho PM, Kenerley CM, Monte E, Baker SE, Grigoriev IV. 2011. Comparative genome sequence analysis underscores mycoparasitism as the ancestral life style of Trichoderma. Genome Biol 12.

3. Druzhinina IS, Chenthamara K, Zhang J, Atanasova L, Yang D, Miao Y, Rahimi MJ, Grujic M, Cai F, Pourmehdi S, Salim KA, Pretzer C, Kopchinskiy AG, Henrissat B, Kuo A, Hundley H, Wang M, Aerts A, Salamov A, Lipzen A, LaButti K, Barry K, Grigoriev I v., Shen Q, Kubicek CP. 2018. Massive lateral transfer of genes encoding plant cell wall-degrading enzymes to the mycoparasitic fungus Trichoderma from its plant-associated hosts.
PLoS Genet 14:e1007322.

4. Baroncelli R, Zapparata A, Piaggeschi G, Sarrocco S, Vannacci G. 2016. Draft whole-genome sequence of *Trichoderma gamsii* T6085, a promising biocontrol agent of Fusarium head blight on wheat. Genome Announc 4.

5. Kubicek CP, Steindorff AS, Chenthamara K, Manganiello G, Henrissat B, Zhang J, Cai F, Kopchinskiy AG, Kubicek EM, Kuo A, Baroncelli R, Sarrocco S, Noronha EF, Vannacci G, Shen Q, Grigoriev I v., Druzhinina IS. 2019. Evolution and comparative genomics of the most common Trichoderma species. BMC Genomics 20:1–24.

6. Studholme DJ, Harris B, le Cocq K, Winsbury R, Perera V, Ryder L, Ward JL, Beale MH, Thornton CR, Grant M. 2013. Investigating the beneficial traits of *Trichoderma hamatum* GD12 for sustainable agriculture-insights from genomics. Front Plant Sci 4:258.

7. Yang D, Pomraning K, Kopchinskiy A, Aghcheh RK, Atanasova L, Chenthamara K, Baker SE, Zhang R, Shen Q, Freitag M, Kubicek CP, Druzhinina IS. 2015. Genome sequence and annotation of *Trichoderma parareesei*, the ancestor of the cellulase producer *Trichoderma reesei*.
Genome Announc 3:885–900.

8. Martinez D, Berka RM, Henrissat B, Saloheimo M, Arvas M, Baker SE, Chapman J, Chertkov O, Coutinho PM, Cullen D, Danchin EGJ, Grigoriev I v., Harris P, Jackson M, Kubicek CP, Han CS, Ho I, Larrondo LF, de Leon AL, Magnuson JK, Merino S, Misra M, Nelson B, Putnam N, Robbertse B, Salamov AA, Schmoll M, Terry A, Thayer N, Westerholm-Parvinen A, Schoch CL, Yao J, Barbote R, Nelson MA, Detter C, Bruce D, Kuske CR, Xie G, Richardson P, Rokhsar DS, Lucas SM, Rubin EM, Dunn-Coleman N, Ward M, Brettin TS. 2008. Genome sequencing and analysis of the biomass-degrading fungus *Trichoderma reesei* (syn. *Hypocrea jecorina*). Nature Biotechnology 2008 26:5 26:553–560.

9. Koike H, Aerts A, Labutti K, Grigoriev I v., Baker SE. 2013. Comparative genomics analysis of *Trichoderma reesei* strains. Industrial Biotechnology 9:352–367.

10. Chung D, Kwon YM, Yang Y. 2021. Telomere-to-telomere genome assembly of asparaginase-producing *Trichoderma simmonsii*.
BMC Genomics 22:1–18.

11. Li W-C, Lin T-C, Chen C-L, Liu H-C, Lin H-N, Chao J-L, Hsieh C-H, Ni H-F, Chen R-S, Wang T-F. 2021. Complete genome sequences and genome-wide characterization of Trichoderma biocontrol agents provide new insights into their evolution and variation in genome organization, sexual development, and fungal-plant interactions. Microbiol Spectr 9. doi: 10.1128/Spectrum.00663-21
12. Li Z, Liu T. 2022. The highly contiguous genome resource of *Trichoderma semiorbis* FJ059, a biological control agent for litchi downy blight. Phytopathology 112:1391–1395.
13. Zhou Y, Wang Y, Chen K, Wu Y, Hu J, Wei Y, Li J, Yang H, Ryder M, Denton MD. 2020. Near-complete genomes of two Trichoderma species: A resource for biological control of plant pathogens. Molecular Plant-Microbe Interactions 33:1036–1039.
14. Tamizi AA, Mat-Amin N, Weaver JA, Olumakaiye RT, Akbar MA, Jin S, Bunawan H, Alberti F. 2022. Genome sequencing and analysis of Trichoderma (Hypocreaceae) isolates exhibiting antagonistic activity against the papaya dieback pathogen, *Erwinia mallotivora*. Journal of Fungi 2022, 8:246.
15. Kannan C, Divya M, Rekha G, Barbadikar KM, Maruthi P, Hajira SK, Sundaram RM. 2022. Whole genome sequencing data of native isolates of Bacillus and Trichoderma having potential biocontrol and plant growth promotion activities in rice. Data Brief 41:107923.
16. Gortikov M, Wang Z, Steindorff AS, Grigoriev I v., Druzhinina IS, Townsend JP, Yarden O. 2022. Sequencing and analysis of the entire genome of the mycoparasitic bioeffector fungus *Trichoderma asperelloides* strain T 203 (Hypocreales). Microbiol Resour Announc 11.
17. Davolos D, Russo F, Canfora L, Malusà E, Tartanus M, Furmanczyk EM, Ceci A, Maggi O, Persiani AM. 2021. A genomic and transcriptomic study on the DDT-resistant *Trichoderma hamatum* FBL 587: First genetic data into mycoremediation strategies for DDT-Polluted Sites. Microorganisms 2021, 9:1680.
18. Li WC, Huang CH, Chen CL, Chuang YC, Tung SY, Wang TF. 2017. *Trichoderma reesei* complete genome sequence, repeat-induced point mutation, and partitioning of CAZyme gene clusters. Biotechnol Biofuels 10.
19. Venice F, Davolos D, Spina F, Poli A, Prigione VP, Varese GC, Ghignone S. 2020. Genome sequence of *Trichoderma lixii* MUT3171, a promising strain for mycoremediation of PAH-contaminated sites. Microorganisms 2020, 8:1258.

<u>Supplementary files</u> (Excel spreadsheets)

Table S2. Core *Trichoderma*-regulated tomato shoot transcripts significantly differential in interaction with either *T. virens* Gv29-8 or *T. virens* GvW as compared to the control (mock), log2 fold at least 2.0

Table S3. *Trichoderma*-regulated tomato shoot transcripts significantly differential between *T. virens* Gv29-8 and T. virens GvW, log2 fold at least 2.0

Table S4. Automated annotation of *T. virens* GvW genome assembly

Table S5. Proteins without Trividraft ID and specific to *T. virens* GvW strain

Table S6. Genes preferentially regulated in *T. virens* GvW while interacting with *S. rolfsii*, as compared to *T. virens* GvW interacting with itself

Table S7. Genes preferentially regulated in *T. virens* Gv29-8 while interacting with *S. rolfsii*, as compared to *T. virens* Gv29-8 interacting with itself

Table S8. Gene list and details accompanying Figure S10

Table S9. Complete transcriptomic data for *T. virens* – *S. rolfsii* interactions

Supplementary Figure Legends

Figure S1. Conidiophore morphology. Images, taken at 800x magnification show arrangement of conidiophores and conidia (dark spheres) in the two strains.

Figure S2. Viable *S. rolfsii* cannot be isolated from the interaction zone. Disks from the junction zone of confrontation with the *T. virens* GvW (P) strain (left) or *T. virens* Gv29-8 (Q) strain (right) were inoculated on PDA amended with benomyl, allowing growth only of *S. rolfsii* (center, control *S. rolfsii*).

Figure S3. Colonization of dead sclerotia. *S. rolfsii* sclerotia were killed by dry autoclaving, and tested as a substrate for growth of *T. virens,* as compared to parasitism of live sclerotia as in Figure 3. Upper two rows, live sclerotia; lower three rows, dead sclerotia.

Figure S4. Biocontrol assay. Bean seedlings were grown in soil infested with *S. rolfsii* (Sr control) or with addition of *T. virens* GvW (P) and *T. virens* Gv29-8 (Q) strains. Mean disease score (see Methods) is shown for each treatment; error bars indicate SEM of 25-27 seedlings from a total of six pots for each treatment.

Figure S5. Pie chart representing classification of *T. virens* GvW (P) strain specific genes.

Figure S6. Pie chart representing classification of organisms in *T. virens* GvW (P) strain specific genes.

Figure S7. Pie chart representing classification of *T. virens* Gv29-8 (Q) strains specific genes.

Figure S8. Genes upregulated in *T. virens* GvW x *S. rolfsii* versus *T. virens* GvW x *T. virens* GvW.

Figure S9. Genes upregulated in *T. virens* Gv29-8 x *S. rolfsii* versus *T. virens* Gv29-8 x *T. virens* Gv29-8.

Figure S10. Distinct transcriptomic patterns of GvW and Gv29-8 in interaction with *S. rolfsii.*

A) Top 20 genes upregulated in *T. virens* GvW x *S. rolfsii* versus *T. virens* GvW x *T. virens* GvW. For details and annotation, see **Table S9**.

B) Top 20 genes upregulated in *T. virens* Gv29-8 x *S. rolfsii* versus *T. virens* Gv29- 8 x *T. virens* Gv29-8. For details and annotation, see **Table S9**.

C) Examples of cluster analysis. The full data set (**Table S9**) was filtered to exclude transcripts with base-mean counts less than 20, and clustered with Genesis. Counts were averaged across experiments for each transcriptome, then imported, normalized across each line of the table using the "normalize genes option" under the "adjust" tab, and clustered using the average linkage WPGMA option, hierarchical clustering. For each panel, one ID was chosen from the lists in A, B and used to search the clustering results (magenta-coloured ID number). The clusters were then expanded at successive nodes, to include the additional genes from panels A, B appearing in the same cluster; these are indicated by blue arrows. Each cluster and the relevant genes are colour coded (diamonds) for reference between panels A,B and C.

Figure S11. Time series for selected transcripts of *T. virens* in interaction with *S. rolfsii*. Each time step is 24-36 hours, sampling time adjusted to the same interaction stage to compensate for slower growth rate of Gv29-8. The lines and symbols are colour-coded to indicate the primer pairs detailed in the table below the graph. Dashed lines, Gv29-8; solid lines, GvW. Markers indicate normalized counts from RNASeq, relative to beta-tubulin ID 88010 (**Table S9**). Lines are qRT-PCR data, mean log2 fold ratio of *T. virens* x *S. rolfsii* interactions from control (self x self *T. virens*) interactions, for three independent experiments, signal calculated as ΔCt relative to beta-tubulin. The numbers shown at time point 3 in the plot indicate probability values for Student's t-test comparing the signal (qRT-PCR) for each *T. virens* x *S. rolfsii* interaction to the corresponding *T. virens* x *T. virens* control pair, at the peak of expression.

Figure S12. Genes downregulated in *T. virens* GvW x *S. rolfsii* versus *T. virens* GvW x *T. virens* GvW. Details of the genes may be found in **Table S6**.

Figure S13. Genes downregulated in *T. virens* Gv29-8 x *S. rolfsii* versus *T. virens* Gv29-8 x *T. virens* Gv29-8. Details of the genes may be found in **Table S7**.

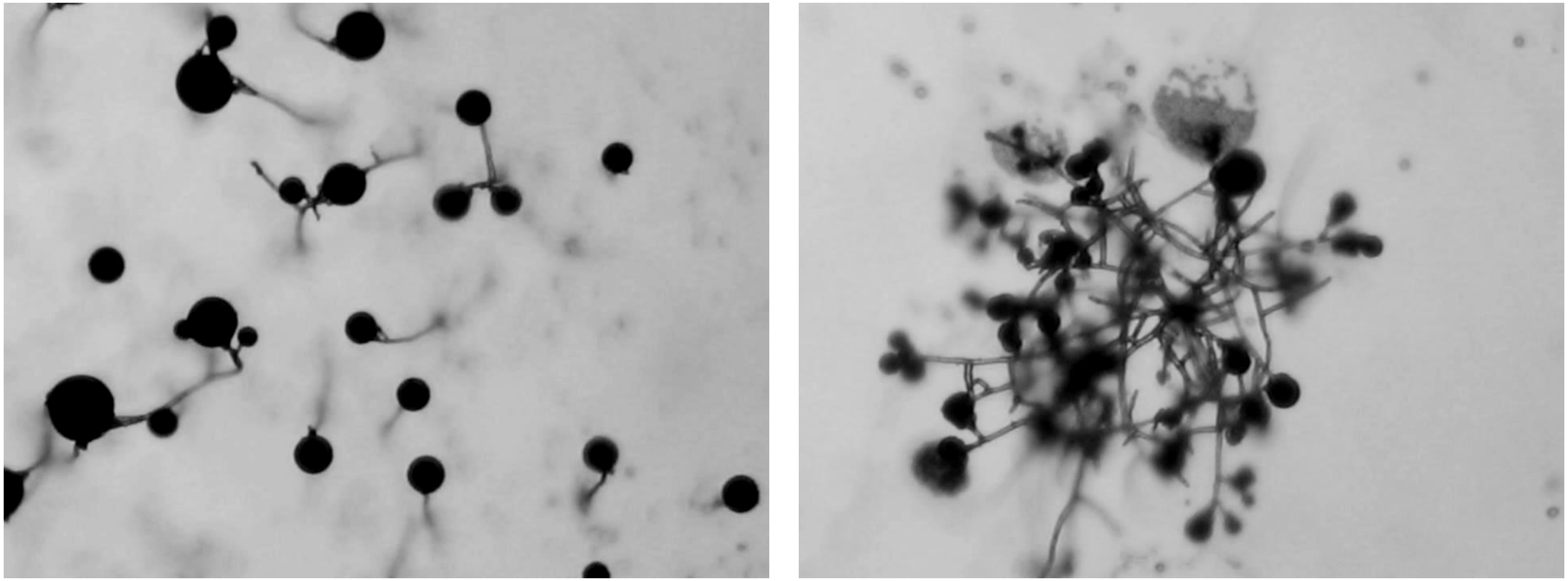

GvW Gv29-8

Figure S1

GvW 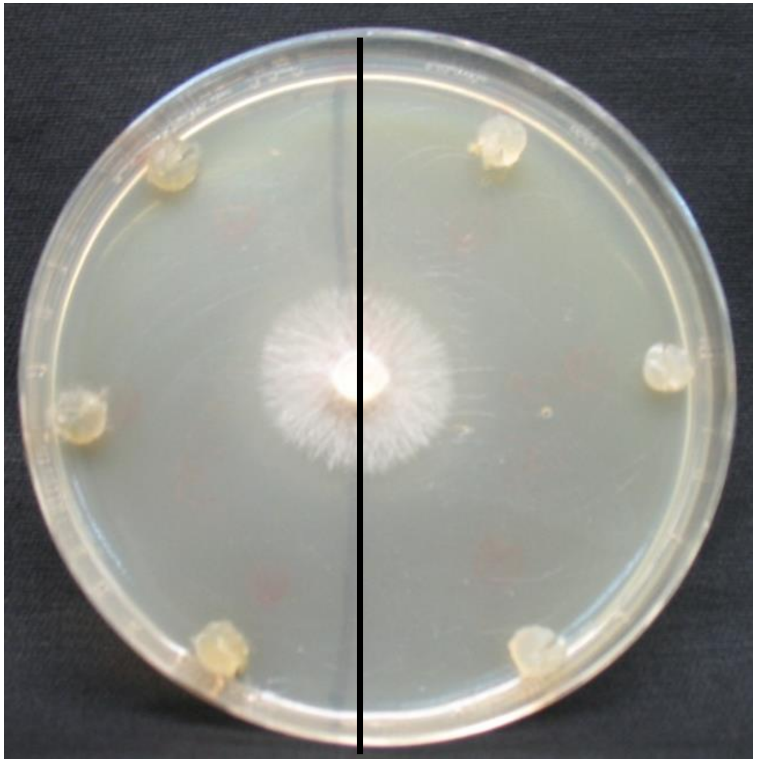 Gv29-8

Figure S2

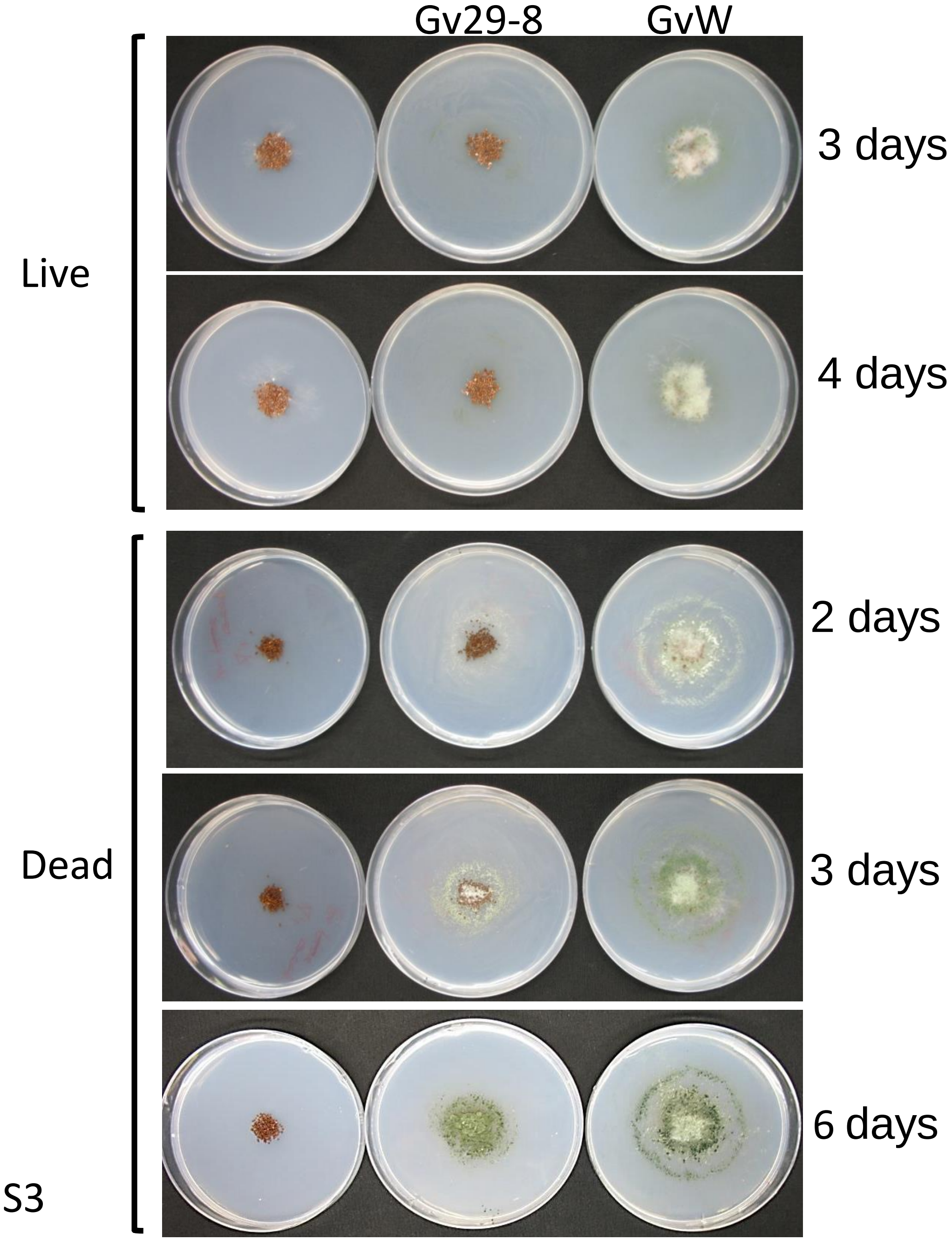

Figure S3

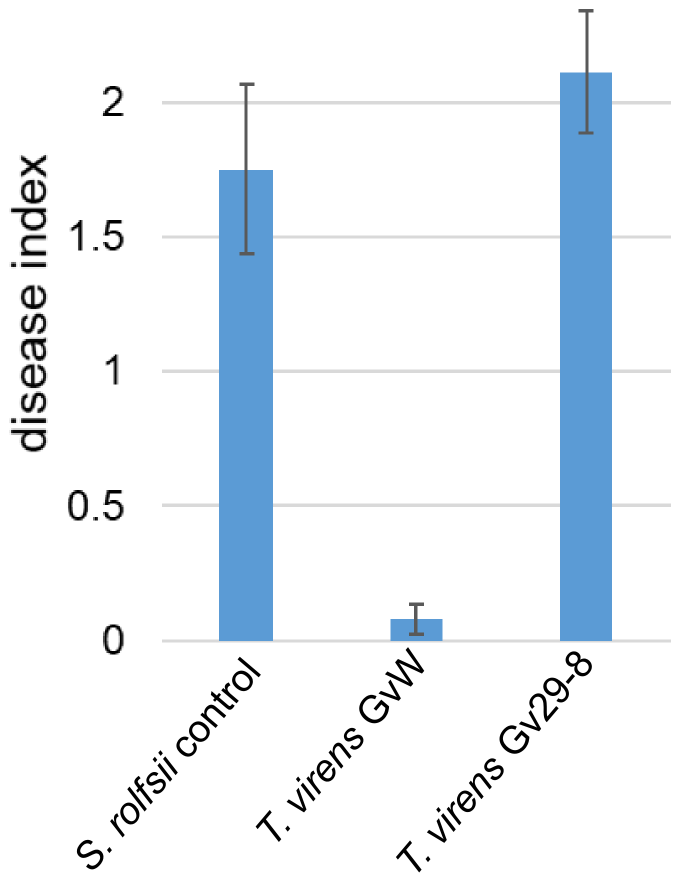
disease index
2
1.5
1
0.5
0
S. rolfsii control
T. virens GvW
T. virens Gv29-8

Figure S4

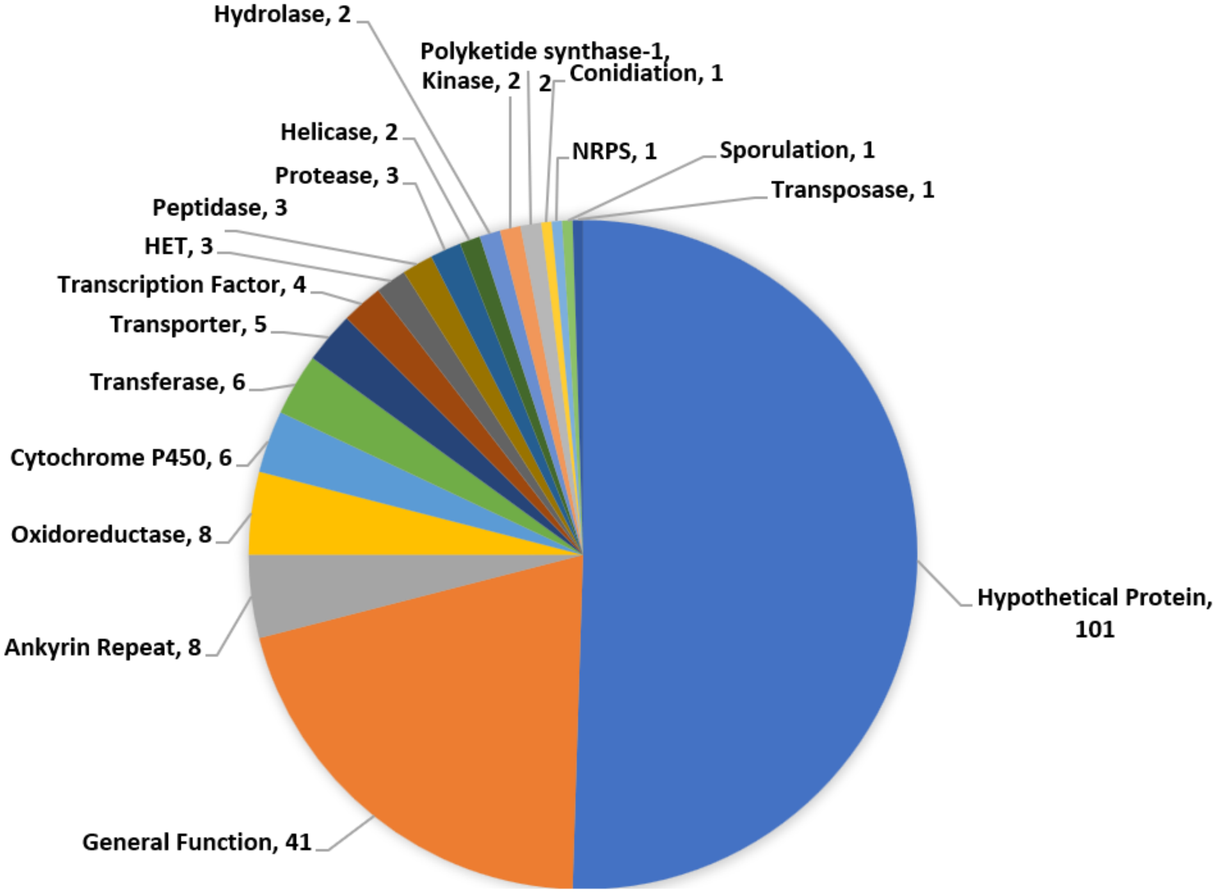
Hydrolase, 2
Polyketide synthase-1, 2
Kinase, 2
Conidiation, 1
Helicase, 2
NRPS, 1
Sporulation, 1
Protease, 3
Transposase, 1
Peptidase, 3
HET, 3
Transcription Factor, 4
Transporter, 5
Transferase, 6
Cytochrome P450, 6
Oxidoreductase, 8
Ankyrin Repeat, 8
Hypothetical Protein, 101
General Function, 41

Figure S5

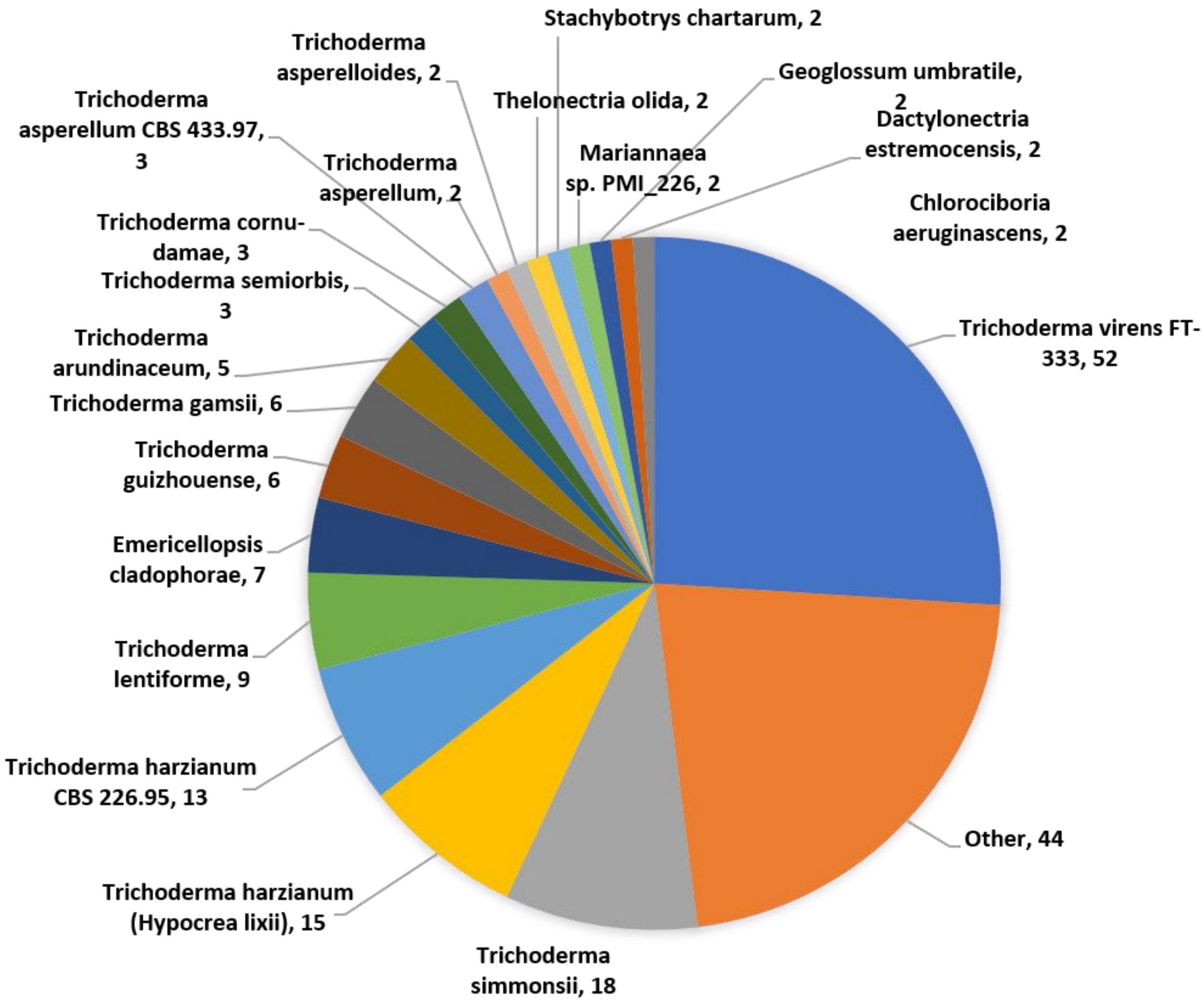
Stachybotrys chartarum, 2
Trichoderma asperelloides, 2
Geoglossum umbratile, 2
Thelonectria olida, 2
Trichoderma asperellum CBS 433.97, 3
Dactylonectria estremocensis, 2
Mariannaea sp. PMI_226, 2
Trichoderma asperellum, 2
Chlorociboria aeruginascens, 2
Trichoderma cornu-damae, 3
Trichoderma semiorbis, 3
Trichoderma virens FT-333, 52
Trichoderma arundinaceum, 5
Trichoderma gamsii, 6
Trichoderma guizhouense, 6
Emericellopsis cladophorae, 7
Trichoderma lentiforme, 9
Trichoderma harzianum CBS 226.95, 13
Other, 44
Trichoderma harzianum (Hypocrea lixii), 15
Trichoderma simmonsii, 18

Figure S6

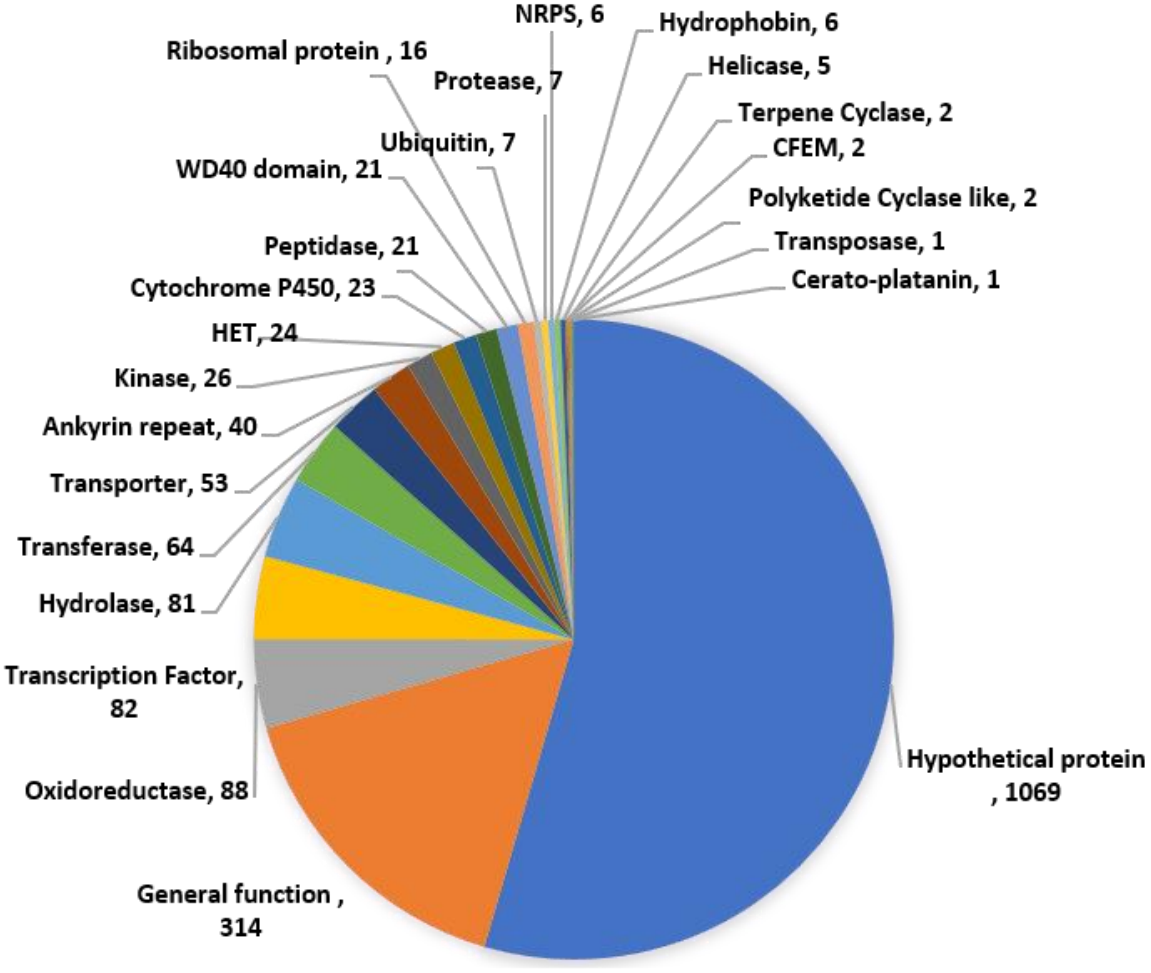
NRPS, 6
Hydrophobin, 6
Ribosomal protein , 16
Helicase, 5
Protease, 7
Terpene Cyclase, 2
Ubiquitin, 7
CFEM, 2
WD40 domain, 21
Polyketide Cyclase like, 2
Transposase, 1
Peptidase, 21
Cerato-platanin, 1
Cytochrome P450, 23
HET, 24
Kinase, 26
Ankyrin repeat, 40
Transporter, 53
Transferase, 64
Hydrolase, 81
Transcription Factor, 82
Oxidoreductase, 88
Hypothetical protein , 1069
General function , 314

Figure S7

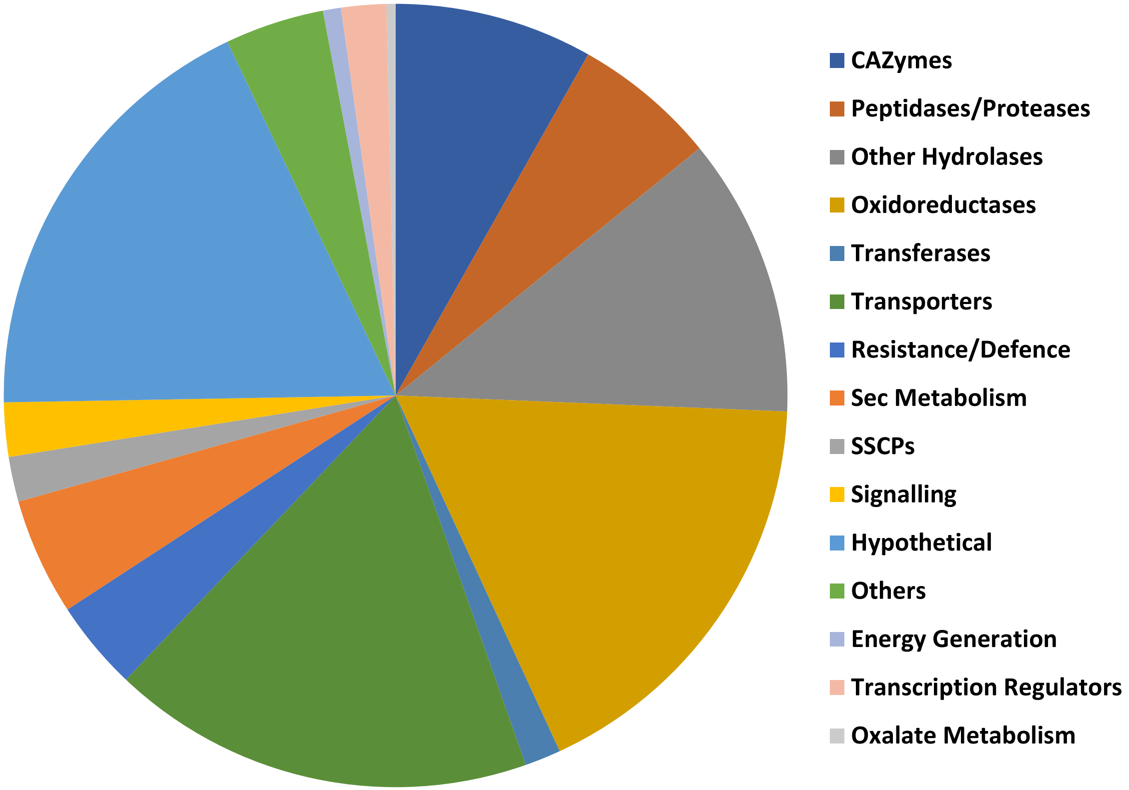

CAZymes	22
Peptidases/proteases	16
Other Hydrolases	31
Oxidoreductases	47
Transferases	4
Transporters	47
Resistance/Defence	10
Sec Metabolism	13
SSCPs	5
Signalling	6
Hypothetical	49
Others	11
Energy generation	2
Transcription Regulators	5
Oxalate Metabolism	1

Figure S8

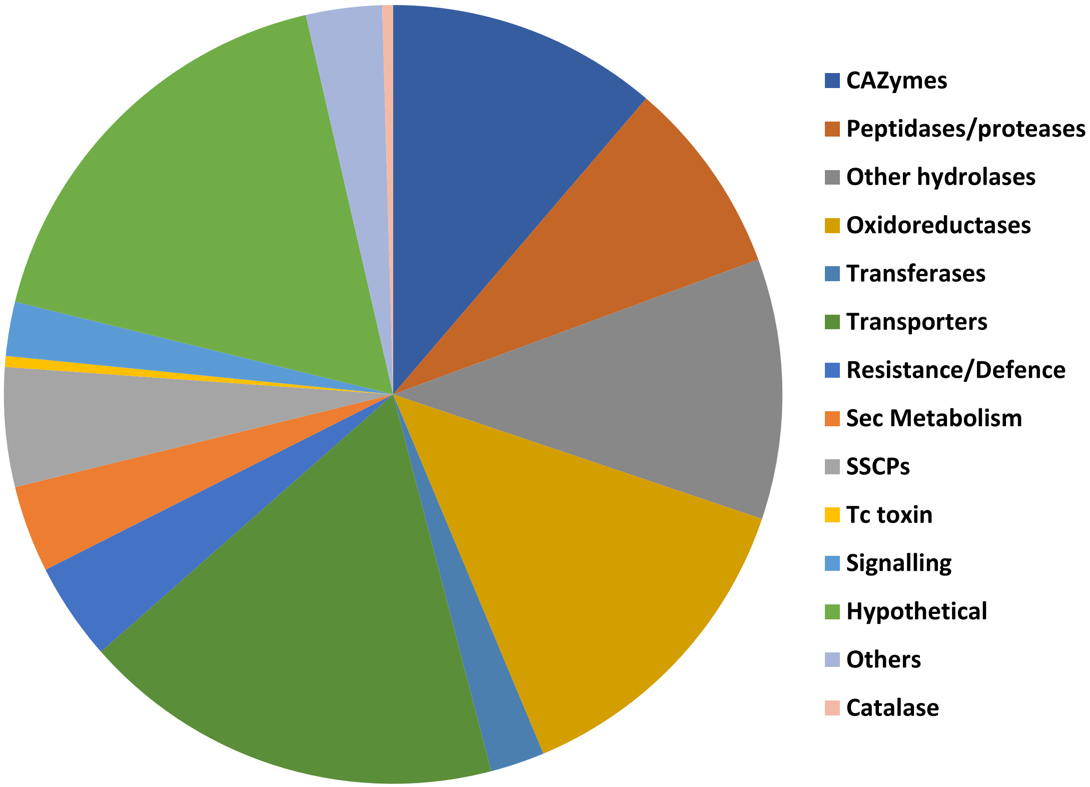

CAZymes	25
Peptidases/proteases	18
Other hydrolases	24
Oxidoreductases	30
Transferases	5
Transporters	39
Resistance/Defence	9
Sec Metabolism	8
SSCPs	11
Tc toxin	1
Signalling	5
Hypothetical	39
Others	7
Catalase	1

Figure S9

A

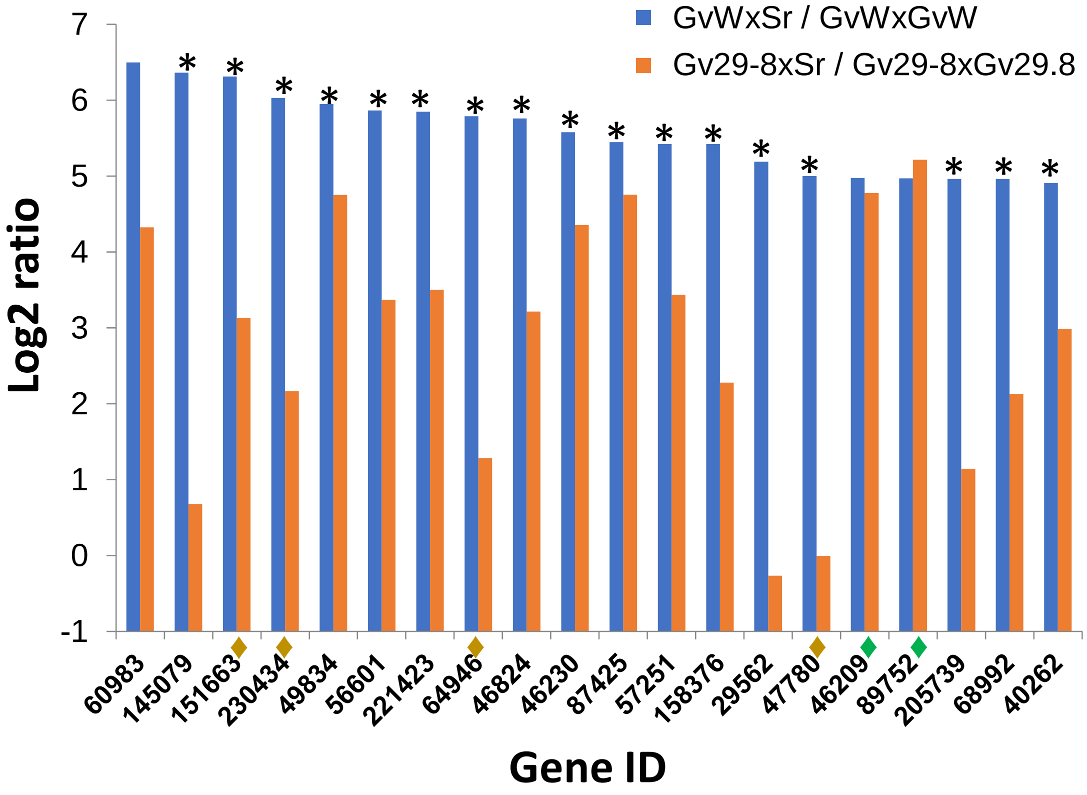

Figure S10A

B

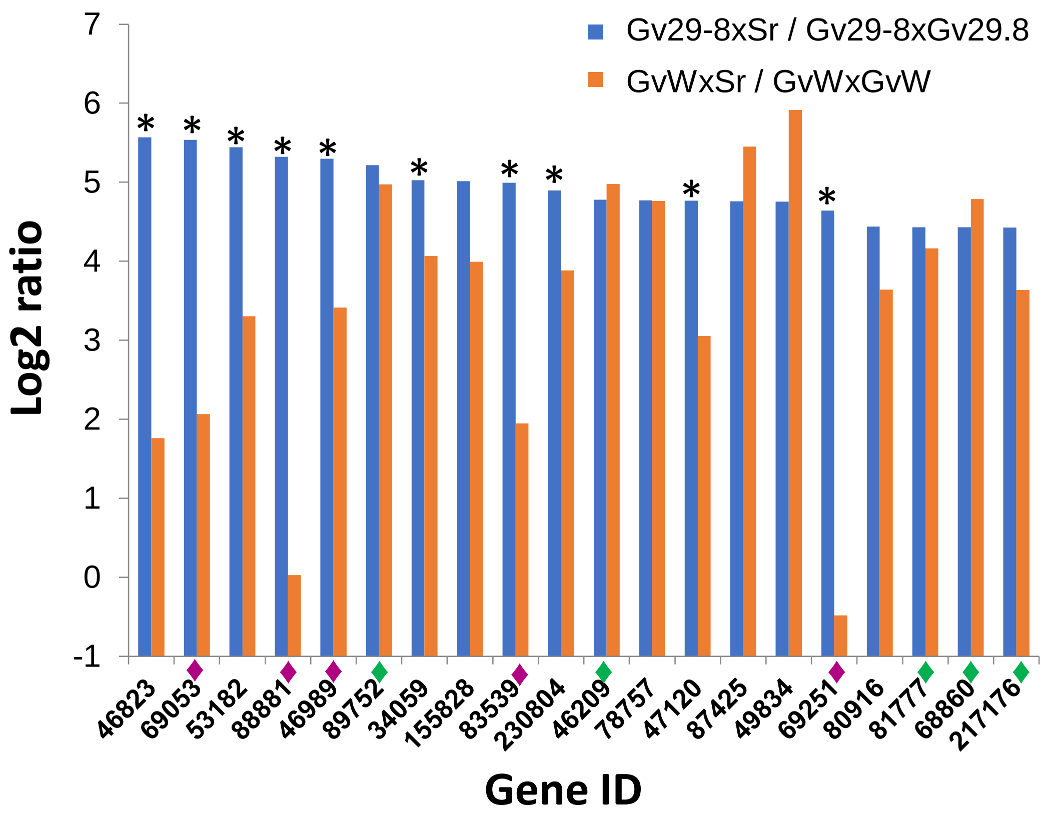

Figure S10B

C

♦ up-regulated PSr and QSr ♦ up-regulated QSr ♦ up-regulated PSr

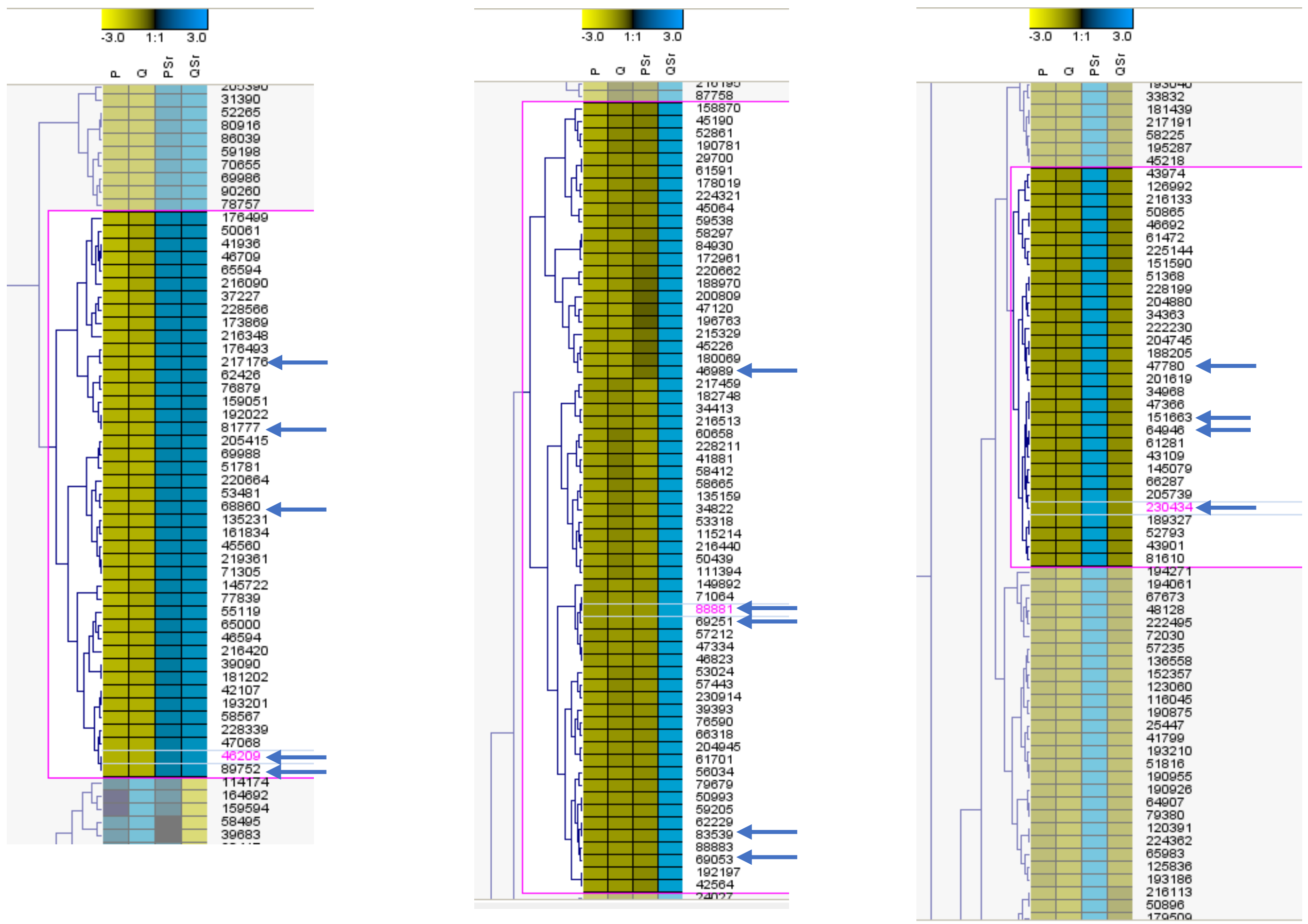

Figure S10C

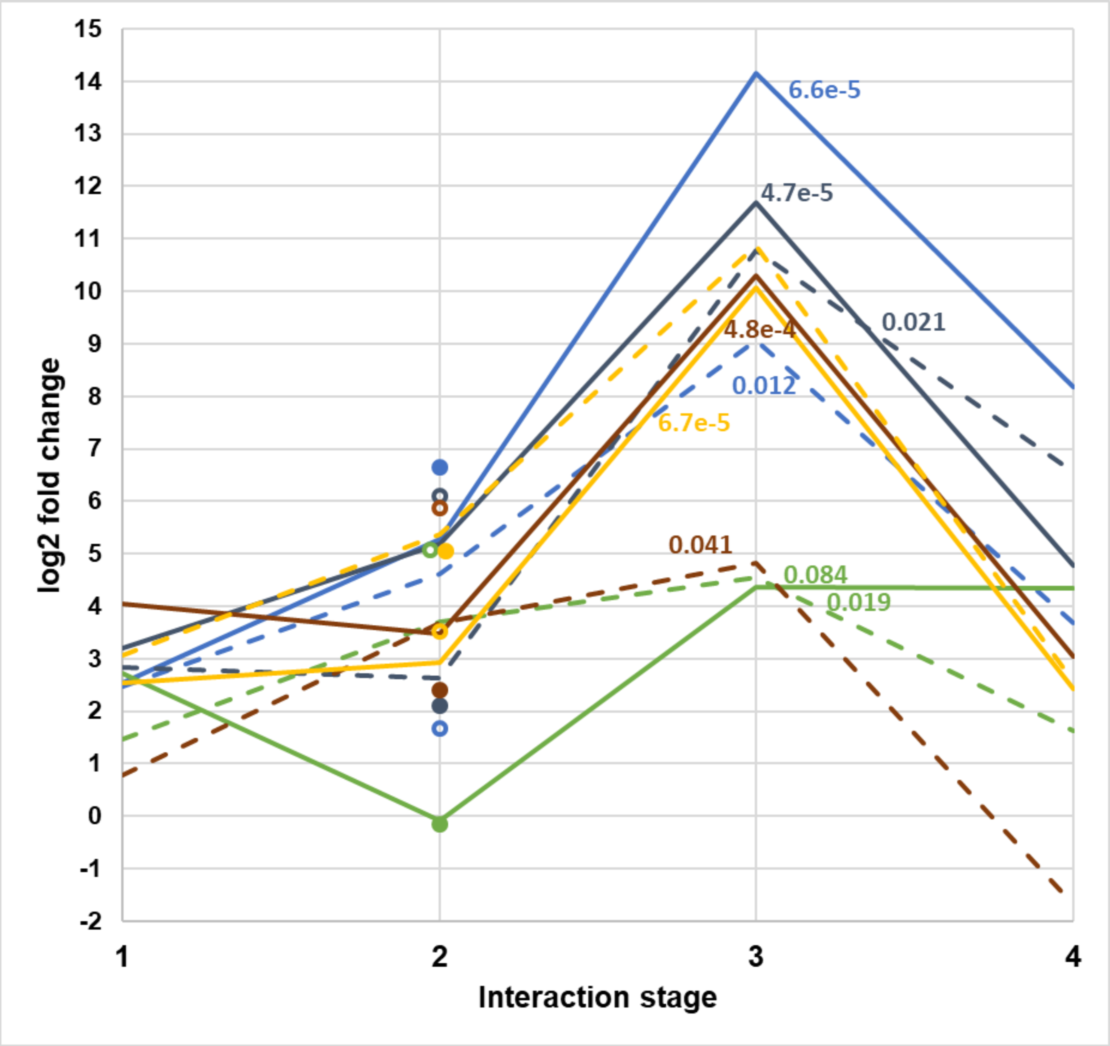

legend	ID	annotation	forward primer	reverse primer
	69251	small secreted cysteine rich protein	GGTAATAGCACGAGGTCGCCAGGCCATATCG	GACGGTGGTGAAGTTTTCGCTATGCGTTGGGC
	64946	no clear annotation	GTGCAAAACGAAGGGGCATTCTGTCTCCATATCC	GAAGCAACGTGAGTCGTGCTGATATGCTTC
	46823	oxalocrotonate tautomerase	ATTCCACCTCCCTCTACCTGGCCGCTTCGAGCAG	ATTTCTGAAATTCGGGCCTGACCTTCATCTCTTCAAC
	192117	2-Hydroxyacid dehydrogenases	GGCAAGTGGCGCGTTTGGCACAG	CTGGCCGTGAAAGTAAGCGCTCGGAATC
	69053	bicupin, oxalate decarboxylase family	GCTCCCCAGATTCAATCACTCGGTGAGAATAC	CATCGCTAGTGTCCGGAATTGGAGCAAC
Gv29-8 empty symbol, dotted line				
GvW full symbol, solid line				

Figure S11

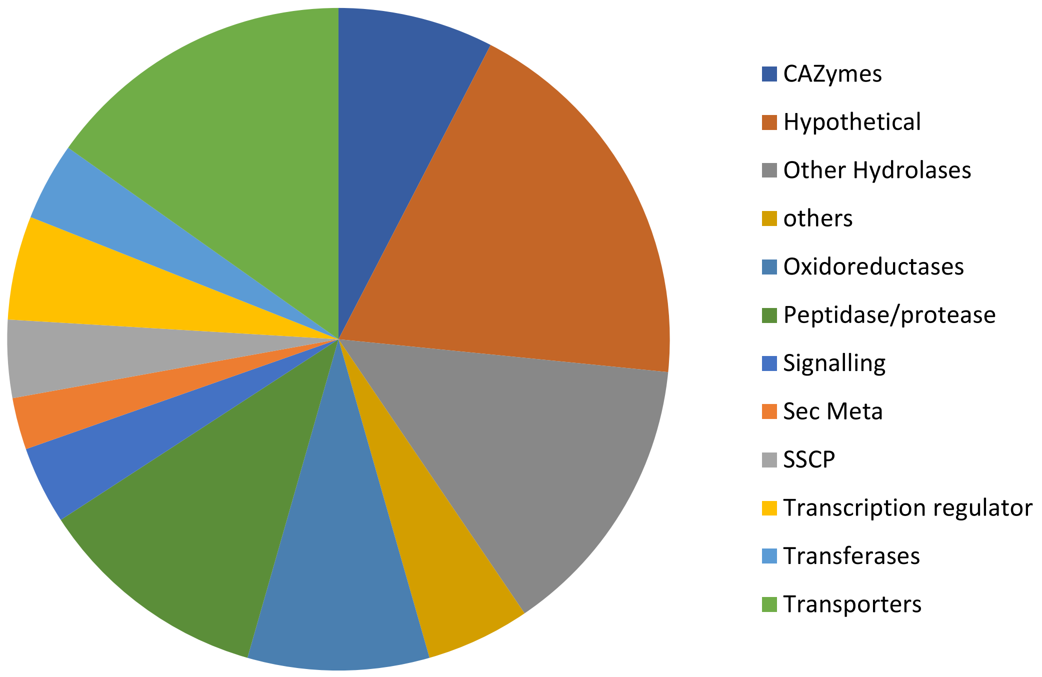

CAZymes	6
Hypothetical	15
Other Hydrolases	11
others	4
Oxidoreductases	7
Peptidase/protease	9
Signalling	3
Sec Meta	2
SSCP	3
Transcription regulator	4
Transferases	3
Transporters	12

Figure S12

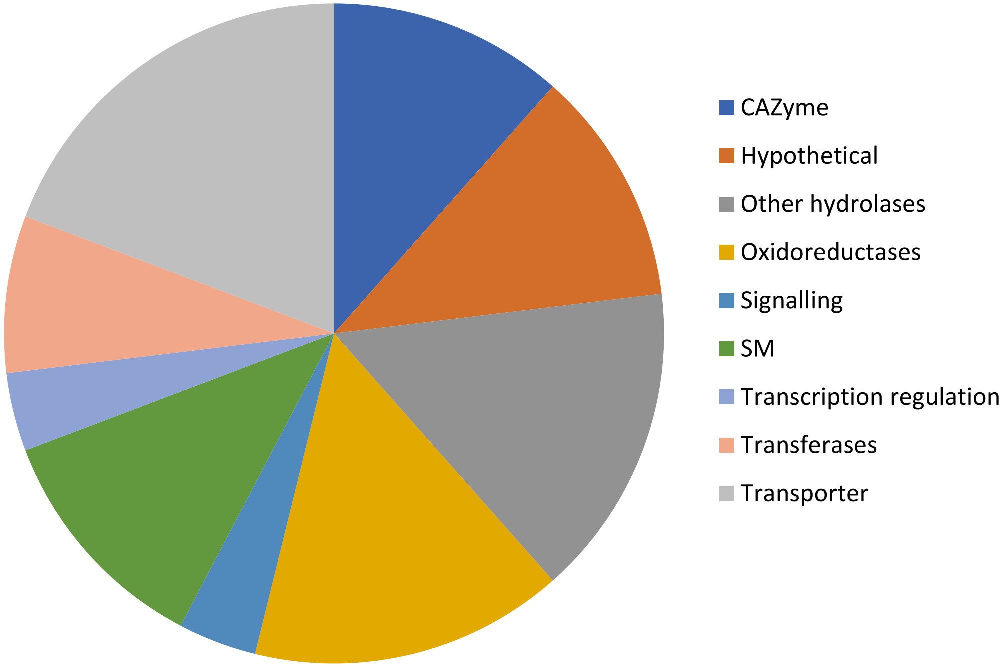

CAZyme	3
Hypothetical	3
Other hydrolases	4
Oxidoreductase s	4
Signalling	1
SM	3
Transcription regulation	1
Transferases	2
Transporter	5

Figure S13
